# Shifting the Balance Between Goals and Habits: Five Failures in Experimental Habit Induction

**DOI:** 10.1037/xge0000402

**Published:** 2018-07

**Authors:** Sanne de Wit, Merel Kindt, Sarah L. Knot, Aukje A. C. Verhoeven, Trevor W. Robbins, Julia Gasull-Camos, Michael Evans, Hira Mirza, Claire M. Gillan

**Affiliations:** 1 Department of Clinical Psychology and Amsterdam Brain and Cognition, University of Amsterdam; 2 Department of Psychology and Behavioural and Clinical Neuroscience Institute, University of Cambridge; 3 Department of Neurochemistry and Neuropharmacology, Institute of Biomedical Research Barcelona; 4 Department of Psychology, New York University; 5 School of Psychology and Global Brain Health Institute, Trinity College Dublin

**Keywords:** habit, outcome-devaluation test, goal-directed action, translational research, reproducibility

## Abstract

Habits are repetitive behaviors that become ingrained with practice, routine, and repetition. The more we repeat an action, the stronger our habits become. Behavioral and clinical neuroscientists have become increasingly interested in this topic because habits may contribute to aspects of maladaptive human behavior, such as compulsive behavior in psychiatry. Numerous studies have demonstrated that habits can be induced in otherwise healthy rats by simply overtraining stimulus–response behaviors. However, despite growing interest in this topic and its application to psychiatry, a similar body of work in humans is absent. Only a single study has been published in humans that shows the effect of extensive training on habit expression. Here, we report five failed attempts to demonstrate that overtraining instrumental behavior leads to the development of inflexible habits in humans, using variants of four previously published outcome devaluation paradigms. Extensive training did not lead to greater habits in two versions of an avoidance learning task, in an appetitive slips-of-action task, or in two independent attempts to replicate the original demonstration. The finding that these outcome devaluation procedures may be insensitive to duration of stimulus-response training in humans has implications for prior work in psychiatric populations. Specifically, it converges with the suggestion that the failures in outcome devaluation in compulsive individuals reflect dysfunction in goal-directed control, rather than overactive habit learning. We discuss why habits are difficult to experimentally induce in healthy humans, and the implications of this for future research in healthy and disordered populations.

Great is the force of habit.

—Cicero (106–43 bc)

The notion that habits powerfully control everyday behavior has attracted attention from philosophers for centuries. Crucial for simplifying our interactions with a complex world, habits allow us to automate actions that we regularly perform. Once formed, habits are thought to be under the control of environmental stimuli, such that they are automatically elicited as opposed to being goal-driven. Only during the last several decades have these intuitive notions of habit become the subject of carefully controlled experimental investigation. Most of this work was conducted in animals.

To study the effect of behavioral repetition on habit formation, researchers have trained rats in operant chambers to obtain food pellets by pressing a lever. Hungry rats will readily learn to do so, and at the surface their behavior certainly looks purposeful or goal-directed. However, to empirically test this, Adams and Dickinson developed the canonical outcome-devaluation test (Adams & Dickinson, 1981). The basic idea behind this paradigm is that a change in the incentive value of an outcome should only directly influence the propensity to perform a previously acquired action if (a) the agent possesses knowledge of the instrumental contingency between the action and the outcome and (b) the agent can evaluate the outcome in light of its current needs and desires and use this evaluation to decide whether to perform the action (Heyes & Dickinson, 1990). In a typical outcome devaluation procedure, the instrumental training phase is followed by a satiety treatment in the home cage (e.g., rats are offered a large amount of food pellets) in order to devalue this food reward. Alternatively, food rewards may be devalued through pairing ingestion with LiCl-induced nausea. Following the devaluation procedure, rats are returned to the operant chamber and are once again offered the opportunity to work to obtain the devalued food reward. This test phase is conducted in extinction to prevent further learning. Using this paradigm, animal researchers have shown that after moderate training, rats will indeed work less for a devalued food reward (i.e., press the lever less frequently) than for a still-valuable food reward. This illustrates that following moderate training, rats are capable of goal-directed, purposeful, control over action. However, following extensive training, rats will persist in lever pressing for the devalued food reward. This indicates that their behavior is no longer under goal-directed control, and is instead automatically triggered by the context/cues, and thus we define the behavior as a *habit*. The induction of habits as a consequence of extensive training was first demonstrated by Dickinson and colleagues (Adams, 1982; Dickinson, Balleine, Watt, Gonzalez, & Boakes, 1995; for a review, see Balleine & O’Doherty, 2010).

To account for the gradual development of behavioral autonomy with extended training, dual-system theories have postulated that repetition shifts the balance between a habit system and a goal-directed system (de Wit & Dickinson, 2009). For example, lever pressing by a hungry rat may initially be controlled by the goal-directed system (de Wit & Dickinson, 2009), but extensive repetition leads to the gradual reinforcement of stimulus–response (S-R) associations in the habit system, through which contextual stimuli in the Skinner box can come to externally control the behavior. Lesioning studies support the dual-system view by providing evidence for dissociable goal-directed and habitual systems in the rodent brain (Balleine & O’Doherty, 2010).

The outcome-devaluation paradigm has only recently been translated to the field of human psychology. Some of these translational studies have adopted the specific-satiety treatment in order to devalue appetitive primary food rewards (Tricomi, Balleine, & O’Doherty, 2009; Valentin, Dickinson, & O’Doherty, 2007; Watson, Wiers, Hommel, & de Wit, 2014) or cigarette rewards in smokers (Hogarth & Chase, 2011). Primary aversive outcomes can also be devalued in the context of avoidance learning paradigms, where for example a shock outcome can be devalued by removing shock delivery electrodes (Gillan et al., 2014). Secondary rewards have also been devalued, through ‘instructed devaluation’ where participants are explicitly informed that one of the behavioral outcomes is no longer desirable (e.g., through currency devaluation; de Wit, Niry, Wariyar, Aitken, & Dickinson, 2007; Gillan, Otto, Phelps, & Daw, 2015). The most frequently used task that uses the instructed devaluation manipulation is known as the slips-of-action task (de Wit, Standing, et al., 2012).

In recent years, research with outcome-devaluation paradigms has been very fruitful for the investigation of individual differences in the balance between habitual and goal-directed control. Neuroscientific investigations have used these tasks to provide evidence for the dual-system view. Functional MRI (fMRI) investigations have implicated the ventromedial prefrontal cortex (vmPFC) in the ability to adjust performance following outcome devaluation through satiation (Valentin et al., 2007) and through instruction (de Wit, Corlett, Aitken, Dickinson, & Fletcher, 2009). A structural MRI study provided evidence that dissociable corticostriatal circuitries correspond to goal-directed and habitual dual systems, in humans as in animals. Estimated white-matter tract strength between caudate and vmPFC was a positive predictor of performance on the slips-of-action task (SOAT), whereas posterior putamen–premotor cortex connectivity was a negative predictor (de Wit, Watson, et al., 2012). Gray matter integrity of the posterior putamen has also been related to habitual performance on this paradigm (Delorme et al., 2016).

Outcome-devaluation tasks have also been used to provide evidence for a shift in balance away from goal-directed control and toward habit in some psychopathologies linked to impaired functioning of corticostriatal functioning. Evidence for this shift was found in obsessive-compulsive disorder (OCD; Gillan et al., 2011, 2014; Gillan, Apergis-Schoute, et al., 2015) Tourette’s syndrome (Delorme et al., 2016), addiction (Ersche et al., 2016; Sjoerds et al., 2013), Parkinson’s disease (de Wit, Barker, Dickinson, & Cools, 2011), and schizophrenia (Morris, Quail, Griffiths, Green, & Balleine, 2015). Moreover, individual differences in self-reported obsessive-compulsive symptoms (Snorrason, Lee, de Wit, & Woods, 2016) and in sensation seeking (Dietrich, de Wit, & Horstmann, 2016) are related to insensitivity to outcome devaluation, as is healthy aging (de Wit, van de Vijver, & Ridderinkhof, 2014). Inconsistent findings were found for obesity (Dietrich et al., 2016; Horstmann et al., 2015; Watson, Wiers, Hommel, Gerdes, & de Wit, 2017), and autism spectrum disorder (Alvares, Balleine, Whittle, & Guastella, 2016; Geurts & de Wit, 2014), and no evidence for failures in outcome devaluation found in anorexia nervosa patients (Godier et al., 2016).

All of the aforementioned studies investigated individual differences in the dual-system balance. Additionally, a couple of outcome-devaluation studies have experimentally induced habits. Dopaminergic and serotonergic manipulations were shown to affect devaluation sensitivity (de Wit, Standing, et al., 2012; Worbe, Savulich, de Wit, Fernandez-Egea, & Robbins, 2015) and experimental stress induction impaired performance on an outcome-devaluation task (Schwabe & Wolf, 2009). However, as most of these studies acknowledge, the paradigms used so far do not allow for separation of goal-directed and habitual processes. This means that either failures in goal-directed control or an excess of the formation of habit might drive failures to adjust performance after devaluation—and this might reasonably differ across the experiments above. Some studies have attempted to fill in this gap by collecting convergent data using neuroimaging, with for example one fMRI study showing that abnormal caudate activity was associated with devaluation failures in OCD implicating failures in goal-directed learning in this disorder (Gillan, Apergis-Schoute, et al., 2015) and another fMRI study relating reduced vmPFC activity to impaired devaluation performance in alcohol dependence (Sjoerds et al., 2013), whereas an sMRI study related impaired devaluation performance in Tourette syndrome to putamen-motor cortex white-matter connectivity (Delorme et al., 2016).

Crucially, there has been a notable absence of studies demonstrating a decrease in sensitivity to devaluation as a function of behavioral repetition. Can we experimentally boost the habit system in healthy humans by extensively training simple behaviors? Habits arising from extensive behavioral repetition have so far been demonstrated in just one experimental study in humans. In this study of Tricomi et al. (2009), participants learned to press keys to obtain two different snack rewards upon seeing different fractal stimuli on the computer screen. Following instrumental training, each participant was selectively satiated on one of the snacks to devalue this outcome. In the critical test phase, participants could once again press for the two snacks. The researchers found that participants in an extensive (3-day) training group were less sensitive to devaluation than the minimal (1-day) group. In other words, after extensive training, participants persisted in responding for devalued outcomes, which is thought to reflect the formation of an inflexible stimulus-driven habit. Furthermore, this fMRI study showed increasing activation of the posterior putamen as a function of behavioral repetition, adding converging evidence that the habit pathway became increasingly involved (although it is not clear whether this BOLD signal was directly related to behavioral sensitivity to outcome devaluation; Tricomi et al., 2009).

These results are exciting because tracking the gradual transition from goal-directed to habitual behavior in healthy humans would allow one to determine the contributions of the goal-directed and habitual processes to action control separately, rather than just having a measure of their respective balance. For this reason, we have made several attempts over the years to develop an experimental paradigm that can reliably induce habitual performance as a function of extensive training. We present five experiments (conducted from 2011 to 2016), in which we aimed to demonstrate increased habitual performance as a function of behavioral repetition: with two versions of an instrumental avoidance task (Experiment 1A and B), with a slips-of-action task (Experiment 2), and finally two independent studies using the task used by Tricomi and colleagues (2009; Experiment 3A and B).

Each of these tasks was based on key principles from the original outcome devaluation studies in rodents (differences in task designs are summarized in Table 1). Participants are first trained to perform simple instrumental responses in the presence of specific cues to earn reward (or avoid punishment), they then undergo an outcome devaluation stage where rewards (or punishments) are reduced in potency, and then we test how outcome devaluation affects performance of the previously learned behavior. In this outcome devaluation test, no feedback is provided contingent on responding to prevent further learning (i.e., the test is conducted in extinction). Selective responding for the still-valuable outcome therefore reflects the ability to flexibly adjust behavior based on anticipation and evaluation of the instrumental outcomes (goal-directed behavior). In contrast, a failure to do so implies that we have experimentally induced the formation of rigid S-R habits.[Table-anchor tbl1]

## Experiment 1: Overtraining Habits on the Avoidance Task

The present studies sought to test if habits can be experimentally induced by overtraining an instrumental avoidance response. To test this, we used a task in which participants were trained to avoid shock (Experiment 1A) or a loud noise (Experiment 1B). Following avoidance training, one of the outcomes was devalued by removing this threat (by disconnecting shock electrodes), thereby rendering the avoidance response unnecessary.

This task has been used previously to study individual variability in the balance between habitual and goal-directed control in OCD (Gillan et al., 2014). In this study, OCD patients performed at the same level as healthy controls after minimal training (three trials per stimulus), but performed worse than controls after additional, extensive training (another 30 trials per stimulus), as reflected in a higher number of avoidance responses for the devalued shock. These results were suggestive that additional training was required to reveal the relatively faster habit formation of OCD patients relative to the control group. In a follow-up study (Gillan, Apergis-Schoute, et al., 2015), a minimal versus extensive training design was not included. OCD patients and healthy controls all received 40 trials of training per stimulus, and once again the OCD group performed relatively poorly in a subsequent outcome-devaluation test. These previous studies suggested that the avoidance task is a promising paradigm with which to investigate habit formation in humans.

In the present studies, we manipulated the duration of avoidance training between participants, in line with the design employed by Tricomi et al. (2009). One group received a minimal and the other a (relatively) extensive training schedule. If participants continued to make the avoidance response even though it no longer produced an outcome that was valuable to them (i.e., the cancellation of an imminent shock or an aversive loud noise), then behavior was deemed habitual. We predicted that overtraining an avoidance response would lead to greater habit formation as evidenced by a reduction in sensitivity to outcome devaluation.

## Experiment 1A

**Figure 1 fig1:**
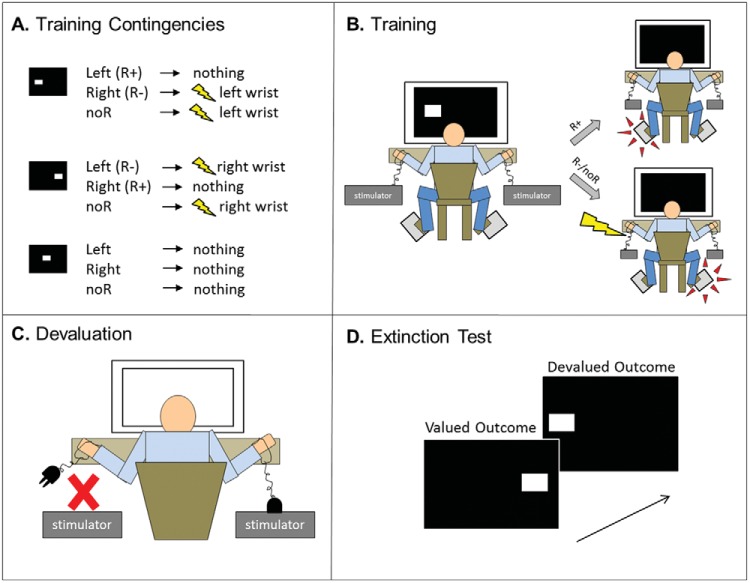
Task design for Experiment 1A. Panel A lists the training contingencies, with the location of a white square against a black background signaling which foot pedal needed to be pressed to avoid an electric shock to the wrist on the corresponding side. A square in the middle of the screen signified that no shock would be delivered. This stimulus was included in the study to measure false alarms. Panel B shows an example of a trial on which the discriminative stimulus signals that the left pedal needs to be pressed to avoid a shock to the left wrist. Panel C shows the outcome-devaluation manipulation consisted of plugging an electrode from one of the wrists out of the stimulator, in clear view of the participant: for half of the participants this was the left and for the others the right wrist. Panel D: During the extinction test, the discriminative stimuli were again shown on the screen in random order while the participants had the opportunity to press the foot pedals. During this brief test, the shocks were no longer delivered regardless of behavior (i.e., the test was conducted in extinction). This prevents new learning during the test phase.

In Experiment 1A, participants were trained to avoid unpleasant, electric shocks to the wrists by pressing foot pedals. For example, as illustrated in Figure 1, Panels A and B, a white square at the left side of the screen signaled that a shock would be delivered to the left wrist, unless the participant pressed the left-foot pedal in time. In contrast, a white square at the right side of the screen meant that participants had to press the right foot pedal to avoid a shock to the right wrist. The 1-day brief training group received two trials of training with each stimulus, and the 1-day extended training group received 33 trials in total. Both groups received all trials consecutively and immediately preceding the outcome-devaluation test.[Fig-anchor fig1]

## Method

### Sample Size Determination

On the basis of an effect size of 0.78 for the interaction between value (valued, devalued) and training duration (1-day training group, 3-day training group) on behavioral responding from Tricomi et al., 2009, it was determined that a total sample size of just 16 (eight per group) was needed to have 80% power to replicate the observed overtraining effect. Note that Tricomi’s original study had a total sample size of 31, with 16 in the 1-day group and 15 in the 3-day group. Aware that small sample sizes can counterintuitively inflate effect size estimates, we conservatively opted for sample sizes of greater than or equal to 24 per group in all studies presented here, which provide approximately 80% power of detecting a smaller interaction effect size (0.40) than would be expected from the prior study. We acknowledge though that the task used by Tricomi and colleagues was markedly different from Experiments 1A, 1B, and 2 and, as such, is a far from ideal benchmark for estimating the required sample size.

### Participants

Seventy-four students at University of Cambridge (43 females and 31 males; *M* ± *SD* age = 20.91 ± 2.15; range = 18–28 years) took part in this study. They were recruited through advertising and paid £10 (equivalent to ~$13.5) for taking part. Ethical approval was received from Cambridgeshire 2 Research Ethics Committee.

### Materials

The experimental task was programmed in ePrime (Software Tools, Inc., Pittsburgh, PA), Version 1.2. Shocks were delivered from two constant current stimulators (DS7A, Digitimer Ltd, Letchworth Garden City, UK), which acted through disposable Ag/AgCl electrodes (Digitimer DENIS10026). Upon arrival, participants completed a standard electric shock work-up procedure to reach a shock level that was “unpleasant or annoying, but not painful” for each wrist. We started the procedure at just 0.2 milliamps and progressively increased the shock level until participants first felt it. Sixty milliamps was the maximum level administered. The final shock intensity selected varied widely across participants (and across wrists), ranging from 0.6 to 60 milliamps (*M* = 8.7, *SD* = 13.3, *Mdn* = 3.4). For some participants, the first time that they felt any stimulation was as high as they wanted to set it. Skin conductance data were also recorded in this session, for which methods and results are presented in the online supplemental material.

### Procedure

Participants were randomly assigned to one of two groups, which were matched for age (*F* < 1), gender (χ^2^ < 1), and handedness (χ^2^ = 1.18, *p* = .28). The 1-day brief group (*N* = 36) received a total of six training trials, two trials per stimulus (left, right, safe), whereas the 1-day extended group (*N* = 38) received 99 training trials, 33 trials per stimulus. Participants were instructed that their task was to avoid receiving shocks. They first received one Pavlovian trial of exposure to each stimulus–outcome pair before beginning their avoidance training sessions.

#### Training phase

Discriminative stimuli were white rectangles, presented on a black background in one of three positions. If the stimulus appeared on the left of the screen, participants were instructed that it signaled that a shock to the left wrist was imminent. Likewise, if the stimulus appeared on the right side of the screen, they were told (on screen) that this meant that a shock to the right wrist was imminent. When the stimulus appeared in the center of the screen, it signaled that no shock would be delivered, and as such this was considered the safe stimulus. To prevent an otherwise imminent shock, we instructed participants that avoidance responses could be made on one of two foot pedals. The left foot pedal could be used to avoid a shock to the left wrist, if executed while the stimulus was displayed on the left of the screen. Likewise, if the stimulus appeared on the right side of the screen, a foot-press on the right pedal would cancel the forthcoming shock to the right wrist. The avoidance response did not terminate the 750-ms discriminative stimulus. If the participant pressed the incorrect pedal to a warning stimulus, or failed to respond within 750 ms, they received a shock. If participants responded to the safe stimulus, nothing happened; this was always safe and was used to measure false alarms. Intertrial intervals (ITI) were 8 s, and the interval between stimulus termination and shock delivery varied randomly between 350 ms and 600 ms. Stimulus presentation order was randomized.

#### Outcome-devaluation test

After training, one of the shock outcomes was devalued by disconnecting the stimulator from the electrodes attached to one of the participants’ wrists. The electrodes attached to the participants’ other wrist remained connected to the other stimulator, and was thus valued. Participants were told that they would no longer receive shocks to that wrist and that their only task was to avoid receiving the remaining shock. Devaluation was counterbalanced for side (right/left) across participants. Participants’ sensitivity to devaluation was then tested in extinction—which meant that no shocks were delivered at all. Participants were not informed of this and so were under the impression that they would still receive shocks to the valued wrist. Participants who have developed habits should show poorer differentiation in their responding toward stimuli associated with valued and devalued outcomes. For example, a perfectly nonhabitual (goal-directed) participant should not respond to the stimulus that predicts the devalued shock outcome, but maintain their responding to the other stimulus to avoid the still-valuable shock. The extinction test consisted of just nine test trials, with three trials per stimulus, presented in a randomized order.

#### Manipulation check

After the test, participants retrospectively rated their confidence that a shock would no longer follow the stimulus that was associated with the disconnected stimulator. The question was as follows: “In the final stage of this experiment, the electrode on your [insert left/right] wrist was removed. Did you believe that the stimulus previously associated with this shock, no longer signaled a shock?” Participants rated on a 100-point visual analog scale (VAS), ranging from 0 (*I thought the stimulus would definitely lead to a shock*) and 100 (*I thought the stimulus would definitely not lead to a shock*). Thus, higher ratings indicated lower expectancy of shock, or greater confidence and understanding of the devaluation procedure. To ensure that all participants had learned the stimulus–response–outcome associations from training, we gave them an explicit test in which they indicated whether each stimulus had predicted a shock and to which wrist.

### Data Analysis

Data were statistically analyzed using SPSS 24.0 and R (IBM Corp., 2015; R Core Team, 2013). Avoidance performance during training was compared with a one-way analysis of variance (ANOVA) using the average percentage of correct responses on the last two trials of training for each discriminative stimulus (i.e., average of left and right accuracy). Only two trials were considered as this is the total number of trials that the 1-day brief group experienced for each stimulus during training. A repeated-measures ANOVA was also used to test if extended training produced stronger habits in our sample. Consistent with the analysis approach from Tricomi and colleagues (2009), the dependent measure was the change in the proportion of (correct) avoidance responses made at the end of training to the extinction (habit) test. Incorrect responses were rare and treated as a nonresponse. Training group (1-day brief, 1-day extended) and value (devalued, valued) were the independent variables. For the crucial interaction between value and group, we complement frequentist statistics with Bayesian model comparison. Specifically, we compare evidence for a main effect model versus one with an interaction between value and group using Bayes factor. Bayesian analyses were conduct in R using the BayesFactor package (R Core Team, 2013).

## Results and Discussion

### Training Phase

**Figure 2 fig2:**
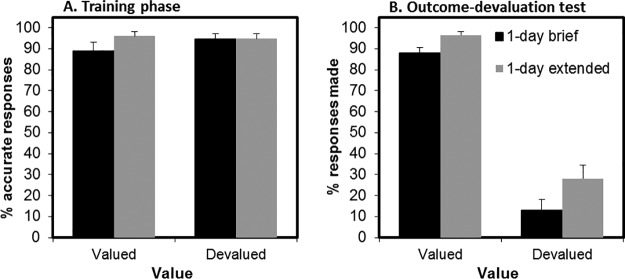
Main results of Experiment 1A. Panel A: The brief (1-day) group received two trials of training per stimulus in total, whereas the extended (1-day) group received 33 trials per stimulus. Shown here are the average accurate response percentages for the final two stimulus presentations in both the 1-day brief (black bars) and 1-day extended training groups (gray bars). Panel B: The critical outcome-devaluation test results are shown in the right panel. Error bars represent standard error of the means.

At the end of training, there was no significant difference in overall performance between the 1-day brief and 1-day extended groups, *F*(1, 72) = 1.25, *p* = .27 (see Figure 2, Panel A). There were also no differences between groups in false alarm responses to the safe stimulus during training (or in extinction; *F* < 1). All participants scored 100% in the questionnaire of explicit knowledge of the stimulus–action–outcome associations.[Fig-anchor fig2]

### Outcome-Devaluation Test

We did not find support for the hypothesis that extensively training avoidance responses produces greater habitual responding as reflected in insensitivity to outcome devaluation (see Figure 2, Panel B). There was a main effect of value on the change in responding from the end of training to the extinction test, *F*(2, 72) = 214.10, *p* < .001, which was driven by the fact that participants reduced responding more for devalued (reduction mean 74%, *SD* = 39%) compared with valued outcomes (reduction mean 2%, *SD* = 22%), consistent with goal-directed control over behavior. The main effect of group was not significant, *F*(1, 72) = 2.30, *p* = .13, and neither was the critical Group × Value interaction, *F*(1, 72) = 1.78, *p* = .19. Bayesian model comparison revealed anecdotal evidence against including the interaction term in the model (Bayes factor = 1.77). Follow-up analyses on raw extinction performance showed that participants in the 1-day extended group responded more than the 1-day brief group toward both valued, *F*(1, 72) = 7.33, *p* = .008, and at trend-level, devalued outcomes, *F*(1, 72) = 3.35, *p* = .071. This suggests that the 1-day brief group might not have reached an equivalent level of baseline performance following their brief (two-trial) training with the task. It must be noted that basic performance differences were not observed in the end-of-training data (reported in the preceding text), thus analysis is based on fewer trials and might therefore be less sensitive.

### Devaluation Manipulation Check

There were no differences between the 1-day brief and 1-day extended training duration groups in their understanding of the devaluation procedure. Their confidence that one of the stimuli from training no longer predicted shock was at the same level (*F* < 1), with average values of 85.97 and 83.32 (*SD*s = 18.97 and 22.05), respectively.

### Summary and Conclusions

Extensive training of an avoidance response did not produce habits as assessed with an outcome devaluation test, a gold-standard methodology translated from classic rodent models. Rather, we saw a general increase in responding for both the valued and devalued outcomes in the 1-day extended training group relative to the 1-day brief group. One possibility is that the 1-day brief training group had insufficient training to reach asymptote in performance, and this performance decrement may have reduced our sensitivity to detect an interaction between group and value. Second, it is possible that the simplicity of the task design, such that stimuli, actions and outcomes engaged the left or right side of the body consistently, made it possible for participants to ignore one side of the screen during the habit test, thereby reducing the potential for habitual slips of action.

## Experiment 1B

To address the issues raised by Experiment 1A, we designed a second study with three levels of training duration (4, 48, 96 trials per stimulus) to test whether extending the duration of training further might reveal a pattern of increasing reliance on habits. We did not include a safe stimulus so that we could maximize the number of training trials per session. Furthermore, instead of presenting stimuli on discrete locations on the screen, we used fractal images and presented them in the center only (as in Gillan et al., 2014). This removed the potential for subjects to divert their attention from one side of the screen as a strategy for avoiding slips of action in the devaluation test. Finally, in contrast to Experiment 1A, here we used an unpleasant sound as the aversive outcome. This was done for practical reasons, including to promote the mobility of the task across labs without dedicated shock equipment.

## Method

The method was almost identical to Experiment 1A, with differences detailed in the following text.

### Participants

Seventy-two participants took part in this study. Participants were 26 females and 46 males with ages ranging from 19 to 37 (*M* = 25.16, *SD* = 4.02). Recruitment was carried out through university and local advertising and participants were paid £10 (equivalent to ~$13.5) for taking part. Ethical approval was gained from the Cambridgeshire 2 Research Ethics Committee.

### Materials

As in Experiment 1A, the experimental task was programmed in ePrime (Software Tools, Inc., Pittsburgh, PA), Version 1.2. The unpleasant noise was delivered through earphones fixed to the exterior of each of the participant’s ears. Upon arrival, participants completed an unpleasant noise work-up procedure to reach a noise level that was “unpleasant or annoying, but not painful.” The sound level administered ranged from 68 to 87 decibels.

### Procedure

Participants were evenly split into three groups of 24, that differed in the duration of training they received. One group received only brief training (1-day brief), another received extended training (1-day extended), and a third group received extended training twice, on two different days (2-day). These groups did not significantly differ in age, *F*(2, 71) = 1.91, *p* = .16, gender (χ^2^ = 5.177, *p* = .08), and handedness (χ^2^ = 2.09, *p* = .35).

#### Setting decibel level for aversive sounds

In contrast to Experiment 1A, here we used an unpleasant sound as the aversive outcome. The decibel level was set for each participant using a work-up procedure identical to that used in studies in which electrical shock is used as the outcome. Specifically, the noise increased incrementally until it reached a level that was “unpleasant or annoying, but not painful.” The sound level administered ranged from 68 to 87 decibels.

#### Practice phase

**Figure 3 fig3:**
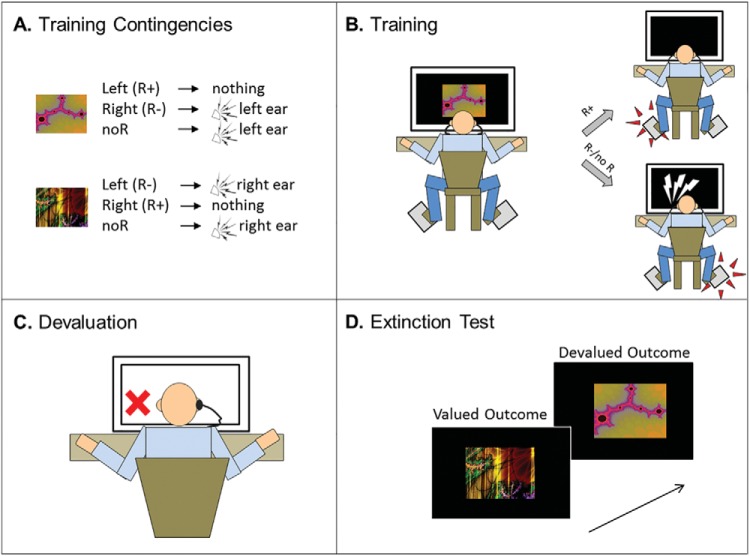
Task design for Experiment 1B. Panel A lists the training contingencies, with two fractal stimuli signaling which foot pedal needed to be pressed in order to avoid an unpleasant noise to the ear on the corresponding side. The S-R assignment was counterbalanced across participants. Panel B shows an example of a trial on which the discriminative stimulus signals that the left pedal needs to be pressed in order to avoid an unpleasant sound to the left ear. Panel C shows the outcome-devaluation manipulation consisted of removing an earpiece from one of the ears, and laying this on the table in front of the participant: for half of the participants this was the left and for the others the right ear. Panel D: During the extinction test, the discriminative stimuli were again shown on the screen in random order while the participants had the opportunity to press the foot pedals. During this brief test, the sounds were no longer delivered.

Following the work-up procedure, participants received Pavlovian exposure to stimulus–outcome pairings and were told that their aim was to avoid receiving unpleasant loud noises. Rather than being discriminable by location, stimuli in this study were two fractals that predicted the unpleasant noise to the left and right ears respectively (see Figure 3, Panel A). Participants received three practice trials per stimulus (six total).[Fig-anchor fig3]

In line with the procedure in Experiment 1A, participants were explicitly instructed that responding on the left pedal would cancel a noise that would otherwise be delivered to the left ear. Likewise, responding on the right pedal would cancel an imminent noise to the right ear. The practice session comprised six trials, three per stimulus type (left, right). Stimulus durations were longer (1,500 ms per stimulus) during this practice phase than for the main experiment (750 ms per stimulus).

#### Training phase

Participants assigned to the 1-day brief group received four trials of training per stimulus. Participants in the 1-day extended group received 48 trials per stimulus. Participants in the 2-day group received 48 trials per stimulus on Day 1 and then returned to the lab within 48 hrs, where they completed a further 48 trials per stimulus.

#### Outcome-devaluation test

Following training, one of the unpleasant noise outcomes was devalued by removing one of the participants’ earpieces and informing them that the stimulus which previously predicted a noise to that ear would no longer lead to receiving an unpleasant noise. The earpiece in the participant’s other ear remained in place, and was thus valued. The earpiece that was devalued was counterbalanced across participants. The habit test was conducted in extinction (no outcomes were delivered) and was identical across the study groups, with all participants receiving 16 test trials in total (eight trials per stimulus).

#### Devaluation manipulation check

Following the habit test, participants retrospectively rated their expectancy that an unpleasant noise would follow the stimulus that was associated with the devalued outcome. This was phrased differently than in the previous study, with participants responding to the following question: “In the final stage of this experiment, the ear-piece on your [insert: left/right] side was removed. Did you believe that the stimulus associated with this side was now safe, and would no longer lead to an unpleasant noise?” Participants rated on a VAS, ranging from 0 (*I thought this stimulus would definitely NOT still lead to a loud noise*) to 100 (*I thought the stimulus would definitely still lead to an unpleasant noise*). Participants also completed an explicit test of stimulus, action, and outcome knowledge. Participants in the 2-day group completed these assessments on day two, following their second session of training.

#### Habituation

Participants rated responses to the question “How uncomfortable/irritating do you find the noise?” on a VAS, ranging from 0 (*not at all uncomfortable/irritating*) to 100 (*extremely uncomfortable/irritating*) at two time points. They rated it prior to beginning avoidance training and at the end of the experiment. This allowed us to measure habituation to unpleasant properties of the noise outcome with increased exposure (results presented in the online supplemental material).

### Data Analysis

Data were statistically analyzed using SPSS 24.0 and R (IBM Corp., 2015; R Core Team, 2013). To test whether there were differences in avoidance performance following different training durations, we averaged the final four trials of each stimulus (left, right) from the training stage and compared this across groups using a one-way ANOVA. Only four trials per stimulus were considered, as this is the total number of trials that the 1-day brief group experienced for each discriminative stimulus during training. A repeated-measures ANOVA was used to test if extended training produced stronger habits in our sample. As in Experiment 1A, the dependent measure was the change in the proportion of (correct) avoidance responses made at end of training to the extinction (habit) test. Incorrect responses were rare and treated as a nonresponse. training group (1-day brief, 1-day extended, 2-day) and value (devalued, valued) were the independent variables. We compared shock expectancy across groups using a one-way ANOVA. We used a repeated-measures ANOVA to examine changes in discomfort as a function of training duration, with time (preexperiment, postexperiment) as a within-participants variable and training group (1-day brief, 1-day extended, 2-day) as the between-participants variable. Frequentists statistics were complement with Bayes factors, where appropriate.

## Results and Discussion

### Training Phase

**Figure 4 fig4:**
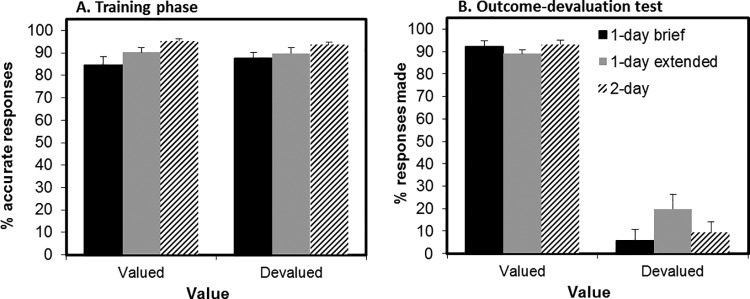
Main results of Experiment 1B. Panel A shows response accuracy of the three groups (1-day brief: white bars; 1-day extended: gray bars; 2-day: black bars) separately for to-be-devalued and to-remain-valuable outcomes. Panel B: The percentage of responses for valued and devalued outcomes during the outcome-devaluation test is shown separately for the three groups. Error bars depict standard error of the means.

At the end of training, there was a significant effect of training group on % correct avoidance responses, *F*(2, 71) = 7.00, *p* = .002 (see Figure 4, Panel A). Pairwise analyses revealed that the 2-day group (*M* = 94.4%, *SD* = 4.8) outperformed the 1-day brief group (*M* = 85.9%, *SD* = 11.6), *F*(1, 47) = 12.81, *p* = .001, and the 1-day extended group (*M* = 90.0%, *SD* = 7.1), *F*(1, 47) = 6.42, *p* = .015. The 1-day extended group did not significantly outperform the 1-day brief group, *F*(1, 47) = 2.44, *p* = .13. All participants scored 100% in the questionnaire of explicit knowledge of the stimulus–action–outcome associations.[Fig-anchor fig4]

### Outcome-Devaluation Test

As in Experiment 1A, there was a significant main effect of value, *F*(1, 69) = 510.1, *p* < .001. Participants decreased responding for the devalued stimulus (reduction *M* = 79%, *SD* = 28), but not for the valued (reduction *M* = −1%, *SD* = 16). The critical Group × Value interaction did not reach significance, *F*(2, 69) = 2.9, *p* = .062, and there was a Bayes factor of just 0.71 (anecdotal) in favor of the inclusion of the interaction. There was no main effect of group on overall responses, *F*(2, 69) = 1.386, *p* = .257. Given that the group by value interaction showed a trend toward significance, we carried out pairwise simple effects to test our a priori hypothesis that increasing training duration would result in failures to reduce responding toward devalued outcomes in stepwise manner.

Comparing the 1-day extended group to the 1-day brief group, we found a significant interaction between value and group, *F*(1, 46) = 5.176, *p* = .028, on the change in responding from training to extinction test. However, tests of simple effects revealed that there were no significant group differences in changes in either responding to valued, *F*(1, 46) = 2.97, *p* = .091, or devalued outcomes, *F*(1, 46) = 1.88, *p* = .177 across groups (see Figure 4). Moreover, when comparing the 1-day brief group with the most extensively trained 2-day group, we did not find any Group × Value interaction (*F* < 1). Similarly, when comparing the 2-day group to the 1-day extended group, we also did not find a significant Group × Value interaction, *F*(1, 46) = 2.39, *p* = .13 (and if anything, participants in the 2-day group showed greater sensitivity to devaluation compared with the less-well-trained 1-day extended group. See the online supplemental material for complementary Bayesian statistics).

### Devaluation Manipulation Check

There were no group differences in shock expectancy ratings between the three training groups (*F* < 1; 1-day brief: *M* = 9.6, *SD* = 19.1; 1-day extended: *M* = 11.8, *SD* = 20.7; 2-day: *M* = 12.6, *SD* = 20.5).

### Summary and Conclusions

In summary, Experiment 1B failed to provide evidence for overtrained avoidance habits, even after doubling the amount of training in the 2-day group. It remains possible that even more extended training would have reduced flexibility. However, it is striking that an eightfold increase in behavioral repetition in group 1-day extended and a 16-fold increase in group 2-day did not have a measurable impact in the studies presented here (see also Gillan et al., 2014). At the very least these findings show that it is nontrivial to experimentally demonstrate a shift in the balance between goal-directed and habitual control over avoidance behavior in healthy participants.

## Experiment 2: Overtraining Habits on the SOAT

The SOAT was developed to pit goal-directed control against habitual control over reward-seeking behavior. This task starts with an instrumental discrimination training session in which different pictorial stimuli predict which response (right or left) will be rewarded with a picture of a reward accompanied with the receipt of points/credits. Assessment of the balance between habitual and goal-directed control, following the training phase, requires that some outcomes are devalued through instruction. This means that participants are instructed that some of the outcomes now lead to the subtraction of points, whereas others are still worth points. Subsequently, participants are shown the discriminative stimuli from the training phase in rapid succession. They should respond when they think the stimulus signals a still-valuable outcome but withhold their response when it signals a devalued outcome. The reasoning behind this SOAT is that strong goal-directed control should lead to good test performance, whereas strong S-R habitual control should lead to slips of action toward no-longer-valuable outcomes. The time pressure that is applied during the test phase should favor habitual performance, rendering this task potentially highly sensitive to reveal habit formation. Furthermore, the test phase is conducted in nominal extinction, which means that participants no longer receive feedback; but participants are instructed that they are still earning/losing points. The use of nominal extinction allows the inclusion of more test trials than are included in a simple extinction procedure. The SOAT has proven its merit by allowing the identification of dissociable corticostriatal circuitries in goal-directed and habitual control (Delorme et al., 2016; de Wit, Watson, et al., 2012) and by providing evidence for reduced sensitivity to outcome devaluation in, for example, addiction and OCD, conditions that are characterized by maladaptive but strongly ingrained repetitive behaviors (Delorme et al., 2016; Ersche et al., 2016; Gillan et al., 2011). However, in all published studies with this paradigm, the length of training was fixed and rather limited.

The question of whether more extended training would shift the balance toward stronger habitual control has not been tested with this paradigm, although one study did use a similar task to investigate the effect of extensive training on action–outcome knowledge, a prerequisite for goal-directed control (de Wit, Ridderinkhof, Fletcher, & Dickinson, 2013). In that study, instrumental discrimination training with four stimuli took place during 6 consecutive days. For each participant, briefly and extensively trained stimuli were presented intermixed during the daily training sessions: Some S-R relationships were extensively trained by repeating them in total 144 times, and other S-R relationships only occurred 36 times. On the 7th day, participants received a test of action–outcome knowledge, in which each trial presented a valuable and a devalued outcome on the screen, and participants were asked to press the key for the still-valuable outcome. This study showed no difference in test performance between minimally and extensively trained stimuli (of the “standard discriminations”). However, as choice behavior was studied in the absence of the stimuli from training, this test does not inform us about habit strength as a function of training duration. The SOAT is more suitable to assess a shift in the balance between goal-directed and habitual control, as here goal-directed control is challenged by learned S-R habits.

Experiment 2 used a within-participant design to compare slips-of-action test performance for minimally trained S-R associations (1-day; 32 repetitions per stimulus) with extensively trained associations (3-day; 160 repetitions per stimulus). We used a within-participant design to cancel out individual variability in habit propensity.

## Method

### Participants

Forty-three participants were tested for this study. There were 32 females and 11 males within the age range of 18 to 31 years (*M* = 21.4, *SD* = 2.7). Recruitment was carried out via the University of Amsterdam website, and participants could choose to receive course credits or a financial compensation of 15 euros (equivalent to ~$17.5). This study was approved by the Ethics committee of the University of Amsterdam.

### Materials

**Figure 5 fig5:**
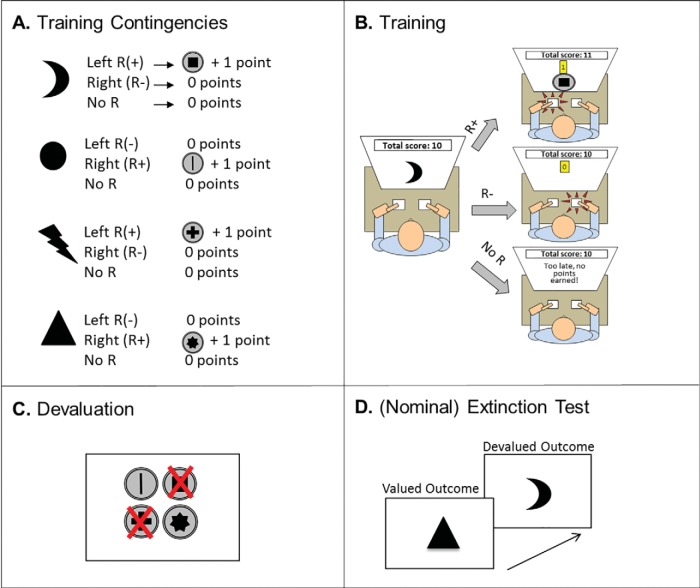
Task Design for Experiment 2. Panel A lists the training contingencies, with four symbols signaling which key needs to be pressed to earn a fictitious coin that was worth points. The stimulus–outcome assignment was counterbalanced across participants; and the pictures used as discriminative stimuli versus outcomes (on the surface of the coins) were reversed for half of the participants. Panel B shows an example of a trial on which the discriminative stimulus signals (the moon symbol) that the left key needs to be pressed to earn a coin with a square symbol. Panel C: The outcome-devaluation manipulation consisted of instructing participants (at the start of each test block) that two coins are still worth points (the still-valuable outcomes), but that the two outcomes with a cross superimposed would now lead to deduction of points from the total score (the devalued outcomes). Across the four test blocks, each outcome was devalued two times. Panel D: During the nominal extinction test, the discriminative stimuli were again shown on the screen in random order, and in rapid succession, while the participants had the opportunity to press the two keys. During this brief test, no outcomes were presented, but participants were instructed that they were still earning (and losing) points, and that their total score would determine their chance to earn cinema tickets at the end of the experiment.

The SOAT was programmed in Visual Basic 6.0. The current task is a simplified version of an earlier design from for example, (de Wit, Standing, et al., 2012). The most important changes are that in the current task there are only two standard discriminations (four stimulus: response–outcome (S:R-O) contingencies), and that the current version uses different pictures as stimuli and outcomes. Most prior versions of the task have used pictures of fruit as stimuli and as outcomes, but in the present study we used pictures of abstract logos instead (see Figure 5, Panel A).[Fig-anchor fig5]

### Procedure

#### Demonstration phase

The session started with a demonstration of the training and test phases of the SOAT (through the use of pictures that were not used in the actual task to preclude learning effects). All instructions were presented on the screen and if necessary paraphrased by the research assistant.

#### Training phase

Before the training phase of the SOAT started, participants were instructed that they could earn pictures of fictitious coins with abstract symbols superimposed on them. The two participants who had won the most coins at the end of the experiment would win two cinema vouchers each. During each training trial, an abstract logo would be presented for maximally 2 s, and participants had to press either a right or left key (i.e., the *M* and *Z* keys on the keyboard) within that time. Participants could learn through trial and error which response led to a rewarding outcome (see Figure 5, Panels A and B). For example, a moon led to a coin with a square and a display of one point, which was displayed for 2.5 s if the correct key was pressed and confirmed by a “cash” sound. In contrast, an incorrect left key press led to a display of zero points and a “buzz” sound. If participants failed to press a key within 2 s, the following warning message was shown: “Too late, you earned no points!”. Four different abstract pictorial stimuli were used, such that each stimulus was consistently paired with either a right or a left key press and with a specific coin. The pictorial logos used as stimuli and as rewards (in the latter case superimposed on the coins) were counterbalanced across participants. The ITI was fixed at 1.5 s.

All participants received training during three consecutive days. They were tested at the same time, with just five participants being tested slightly longer than an hour apart on the different days. During the first two days, they were trained on a single instrumental discrimination (S1:R1-O1; S2:R2-O2). They received 16 blocks of training in total (with a short break in the middle), with each block containing four repeats of each of the two stimuli, amounting to a total number of 128 trials per day. During the third and final day, they received further training on the 3-day discrimination, as well as on an additional 1-day discrimination (S3:R3-O3; S4:R4-O4). Again, there were 16 blocks of training, but this time each of the four stimuli was repeated twice per block. Therefore, the extensively trained stimuli were each presented in total 160 times, and the minimally trained stimuli each 32 times.

#### Outcome-devaluation test

Participants received instructions of a ‘stock market crash’ that meant that not all currencies were valuable anymore. There were four test blocks, that each started with a 10-s devaluation screen that displayed all four currency outcomes, with two outcomes shown with a cross superimposed indicating that these two outcomes had been devalued (see Figure 5, Panel C). There were four possible devalued outcome pairs, as one was always paired with a right-hand response and the other one with a left-hand response. The order in which the four outcome devaluation pairs were presented across the consecutive blocks was randomly determined for each participant. Participants were instructed that responding to a stimulus associated with a devalued outcome would lead to subtraction of coins and therefore a reduced total score. Therefore, responses to these stimuli had to be inhibited. However, participants should continue to respond quickly and correctly for stimuli associated with valued coin outcomes that were still worth points. Following the devaluation screen, the discriminative stimuli were shown in rapid succession (1-s screens, with the ITI fixed at 1 s; see Figure 5, Panel D). The test was conducted in ‘nominal extinction,’ meaning that feedback was no longer provided to preclude further learning, but participants were aware that the accuracy of their responses was still recorded and that they would be told their total score at the end. After completion of the slips-of-action task, participants filled out the contingency knowledge questionnaires, were debriefed, and reimbursed for their participation.

#### Intention formulation

In this experiment, we additionally investigated a “planning technique” manipulation, which turned out to be ineffective and is not related to the hypothesis of the present article. Nonetheless, for completeness, this manipulation is detailed in the online supplemental material.

### Data Analysis

Data were statistically analyzed with SPSS (Version 22.0, IBM Corp., 2015). In the training phase of the SOAT, participants needed to learn by trial-and-error which key to press to each of four different discriminative stimuli. Therefore, there is a clearer S-R learning component here than in the other tasks reported in this article. To inspect the learning curve, we chose to adopt a different approach to the analysis of these training data. Training performance was assessed with a repeated measures ANOVA with as within-participant variables block set (each training day consisted of 16 blocks which were averaged to obtain values for eight block sets), and training duration (stimuli trained for 1 day vs. 3 days). To assess habit formation, a repeated measures ANOVA was conducted on the response percentages during the slips-of-action test with the within-participant variables training duration for stimuli (1-day, 3-day) and outcome value (devalued, valued).

## Results and Discussion

### Training Phase

**Figure 6 fig6:**
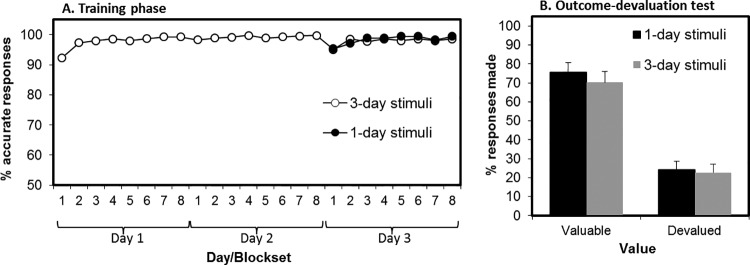
Main results of Experiment 2. The effect of training duration was investigated with a within-participant design. Panel A shows accuracy during the three days of training separately for the extensively trained stimuli (empty dots; 3-day) and for the briefly trained stimuli (filled dots; 1-day). The brief stimuli were each presented 32 times in total during eight block sets, whereas the extensively trained stimuli were each presented 96 times. Panel B: The critical outcome-devaluation test results are shown in the right panel. The black bars represent the 1-day training stimuli, and the gray bars the 3-day training stimuli. Error bars represent standard error of the means.

There was no effect of training duration on performance during Day 3 (*F* < 1), neither was there an interaction with block set (*F* < 1). A main effect of block indicates that participants improved their performance in the course of training during Day 3, *F*(7, 294) = 5.10, *p* < .001 (see Figure 6, Panel A). Paired-samples *t* tests also failed to reveal differences in S-R/R-O/S-O contingency knowledge for the 1-day and 3-day stimuli (*p*s > .18), with average group scores ranging between 1.81 and 2.00.[Fig-anchor fig6]

### SOAT

As can be seen in Figure 6, Panel B, performance on 1-day and 3-day stimulus trials was virtually indistinguishable. Indeed, there was no effect of training duration, *F*(1, 42) = 1.74, *p* = .20, or of Training Duration × Value, *F*(1, 42) = 1.63, *p* = .21. Participants were able to direct responses selectively toward still-valuable outcomes, as confirmed by a significant main effect of value, *F*(1, 42) = 80.62, *p* < .001. Complementary Bayesian analyses revealed moderate evidence against including the interaction between training duration and value in the model, with a Bayes factor of 4.04.

Of the 43 participants tested, it appeared that 6 failed to fully understand the instructions. These participants responded more for devalued than for valuable outcomes in either the extensive/minimal condition or both and/or failed to respond at all for valuable outcomes in either condition. Excluding these 6 participants from the analysis did not affect the pattern of results (Training Duration × Value: *F* < 1).

### Summary and Conclusions

We failed to find evidence for an effect of extensive training on behavioral sensitivity to outcome devaluation. These results show that simply increasing behavioral repetition in the SOAT does not necessarily result in behavioral inflexibility. One key issue might be the abstract nature of the instructed devaluation procedure and the reinforcers used; it is possible that this kind of devaluation is more dependent on goal-directed processing and this overrides any influence of stimulus-response repetition. We address this and other potential limitations of this design in two final studies.

## Experiment 3. Replication Attempts of Previous Overtraining Study (Tricomi et al., 2009)

To date, only one experimental paradigm has provided evidence that habits can be experimentally induced in healthy humans using overtraining (Tricomi et al., 2009). In the training stage of this experiment, different fractal stimuli on the computer screen signaled that left and right key presses would be rewarded with two different food rewards: M&Ms^®^ (Mars, McLean, Virginia) and Fritos^®^ (Frito-Lay Inc., Plano, Texas). One group of participants received brief 1-day training (a single session), whereas another 3-day group was trained extensively during three consecutive days (six sessions). Subsequently, one of the food rewards was devalued through specific satiety. In the final critical extinction test, participants were once again offered the opportunity to press the keys during exposure to the fractal stimuli from training. In line with studies in rodents, Tricomi et al. (2009) demonstrated in humans that briefly trained behavior is sensitive to current outcome value, whereas extensively trained behavior persists despite devaluation of its outcome.

There could be several reasons why the paradigm used by Tricomi and colleagues (2009) might be more sensitive to a shift in the dual-system balance than the tasks reported in Experiments 1 and 2. First, a variable interval (VI) schedule was employed as opposed to a fixed ratio schedule. Animal research has shown that interval schedules reduce sensitivity to outcome devaluation relative to ratio schedules (Dickinson, Nicholas, & Adams, 1983). Furthermore, specific satiety may offer a more gradual and less explicit devaluation procedure than the removal of the electrodes/earplugs in Experiment 1 and the instructed devaluation manipulation in Experiment 2, thereby reducing conscious attempts to engage goal-directed control. Finally, in successful experimental demonstrations of habits in rats, the animals were trained to perform a single response to obtain a single outcome (Adams, 1982). In contrast, in Experiments 1 and 2, participants could always choose which key to press. The paradigm of Tricomi and colleagues is more similar to the animal models in the sense that only a single response is signaled to be available on each trial.

Therefore, we carried out two independent replication studies using the procedure from Tricomi et al., 2009, one study was carried out at New York University (Experiment 3A) and the other at the University of Amsterdam (Experiment 3B). The study designs and procedures matched the information provided in the methods section of Tricomi et al., 2009. Where information was not readily available, it was solicited and received from the lead author of the original study. We describe this procedure in detail herein, along with minor divergences from the original study. The sole major difference between both of these studies and the original study is that our experiments were not conducted inside an fMRI scanner.

## Method

### Participants

Sixty-four healthy participants participated in the experiment (40 females, 24 males; *M* ± *SD* age 22.45 ± 3.99; range = 18–35 years). Participants were prescreened using an online questionnaire prior to being scheduled to make certain that they were not actively dieting and therefore enjoyed eating M&Ms and Fritos. Other exclusion criteria were scores <20 on the eating attitudes test (Garner, Olmstead, Bohr, & Garfinkel, 1982) and any allergies/dietary restrictions that would prevent them from consuming a range of candy bars and potato chips. Participants were required to fast for 6 hr prior to each experimental session; water was allowed during the fasting period. Participants received $10/hr compensation. All participants gave written informed consent and the study conforms with the Code of Ethics of the World Medical Association (Declaration of Helsinki, Rickham, 1964). This study was approved by the New York University Committee on Activates Involving Human Subjects.

### Materials

The experimental paradigm was programmed in MATLAB Psychtoolbox (Mathworks, Natick, MA). Food rewards were M&Ms and Fritos. To test a secondary hypothesis (that was predicated on the successful induction of habits in the 3-day group), we included several other measures before and after the habit task. These measures are detailed in the online supplemental material.

### Procedure

Participants were randomly assigned to one of two groups: the 1-day group (*N* = 33) or the 3-day group (*N* = 31). These groups were matched for age, *t*(62) = 1.00, *p* = .32; with averages of 21.97 (*SD* = 4.41 and 22.97 [*SD* = 3.52], respectively), body mass index (BMI), *t*(62) = .60, *p* = .55; with averages of 24.10 (*SD* = 4.61 and 23.37 [*SD* = 5.27], respectively), and gender (χ^2^ < .10, *p* = .75).

#### Training phase

**Figure 7 fig7:**
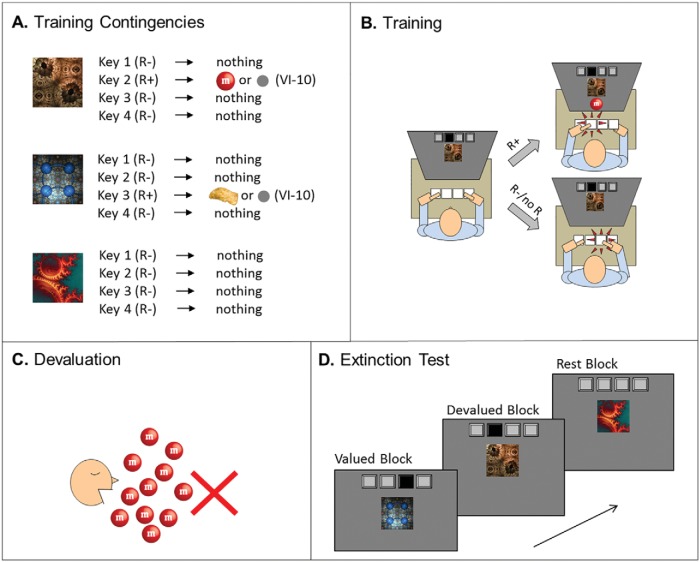
Task design for Experiment 3A and 3B. Panel A lists the training contingencies, with two fractal stimuli signaling which key needed to be pressed (on a variable interval [VI]-10s schedule) to earn M&Ms and Fritos. The S-R assignment was counterbalanced across participants. Panel B shows an example of a trial on which the discriminative stimulus signals that the left key needs to be pressed to earn M&Ms. Panel C: The outcome-devaluation manipulation consisted of satiating participants on one of the two food rewards: For half of the participants these were the M&Ms and for the other half the Fritos. Panel D: During the extinction test, the three discriminative stimuli (corresponding to rest, devalued and valued blocks) were again shown on the screen in random order while the participants had the opportunity to press the keys. During this 3-min test, responding no longer resulted in the food outcomes.

The experimental design was based on that of Tricomi et al. (2009; see Figure 7). Each session comprised 20 blocks in total; 12 task blocks (six Frito, six M&M) and eight rest blocks, each lasting for either 20 s or 40 s. Sixteen blocks were of a 20-s duration, and four lasted 40 s; but in a slight deviation from the Tricomi et al. (2009) design, the duration for a given block was randomly determined (in the Tricomi et al., 2009 design, rest blocks were always 20 s). Block order was pseudorandomized, with trial sequences generated independently for each participant with the condition that participants not receive the same block type twice in a row. In each block, a fractal was displayed in the center of the screen, below four gray squares, which corresponded to each of the four available keyboard buttons. One of these squares was highlighted on the screen during each block, indicating that this button was active. These highlighted buttons were consistently paired with one of the fractals (e.g., Fractal A was always linked to the leftmost button), and this did not change across sessions or days. Each fractal was linked to an outcome; one fractal signaled that M&Ms were available on that trial, another signaled that Fritos were available, and a third indicated that the current block was a rest block. In rest blocks, no buttons were highlighted. At the beginning of each day, participants were provided detailed instructions and completed a short practice session (details are provided in the online supplemental material). The instructions included the following statement:

You can press the button as often or as little as you like. If you do not want any more Fritos or M&Ms, you don’t have to continue pressing; otherwise you should try to earn as much reward as possible. You should pay attention to which fractals go with which responses and rewards.

Note that in the original study (Tricomi et al., 2009), participants completed instructions and a practice session on Day 1 but did not repeat this at the beginning of each day (E. Tricomi, personal communication, May 5, 2017). If participants asked for clarification at any point during the experiment, they were told “You can press the button as often or as little as you like.” The reward schedule for button presses was governed by a VI-10 schedule. This meant that the reward became available on average every 10 s. If a button press was rewarded, a picture of the food appeared on the screen. If a button press was not rewarded, a gray circle appeared each time they pressed. At the end of each day, participants learned how many of the two food items they had won and were given an amount of that food to eat, that was proportional to what they earned during the prior session (33% of total earnings for each food). Participants were also offered a glass of water. The 1-day group completed two sessions, each lasting 8 min. The 3-day group completed four sessions, each also lasting 8 min, on each of 3 consecutive days.[Fig-anchor fig7]

#### Outcome-devaluation test

After the final training session was complete (on Day 1 for the 1-day group and Day 3 for the 3-day group) and participants had consumed their winnings, a devaluation procedure was conducted. Participants were given a “bonus.” The screen displayed the following: “All you can eat [food],” where food was either Fritos or M&Ms. They were told by the experimenter to eat the food until it was no longer pleasant to them. Unlike in the study of Tricomi et al. (2009), further clarification was provided at the participants’ request: “Eat the food until you do not want anymore” or “If I gave you more [food], would you eat it?” Participants were presented with a bowl of the bonus food, which was refilled by the experimenter gradually as they ate. If participants finished eating, they were asked the following: “If I gave you more [food], would you eat it?” If they answered “Yes,” their bowl was refilled until they answered “No.” This devaluation procedure served to selectively reduce the value of one of the food outcomes from training (i.e., devalued), whereas leaving the other food type still valuable (valued). This devaluation procedure was identical to that reported in Tricomi et al. (2009) and supplemented by personal communications with Dr. Tricomi (August 29, 2014 and September 23, 2014).

After the devaluation procedure was complete, participants in both groups completed the 3-min devaluation test. This test was conducted in extinction, meaning that no rewards were delivered. Consistent with Tricomi et al. (2009), there were three valued blocks, three devalued blocks, and three rest blocks, each having a 20-s duration. Block order was again pseudorandomized so that block type did not repeat twice in a row. If participants continue to respond when presented with the fractal that was linked to the devalued food, then this is evidence that a habit has been formed. If participants selectively reduce their responding toward the devalued food, then their behavior is under goal-directed control.

#### Manipulation check

At the beginning of Day 1 and after the experiment was complete (Day 1 for the 1-day group and Day 3 for the 3-day group), participants provided self-report ratings. The rating questions were as follows: For hunger: “How hungry are you right now?” (rated on a 0 to 20 scale, ranging from *very full* to *very hungry*); for Fritos and M&Ms, specifically and separately: “How pleasant do you usually find this type of food?” (rated on a 0 to 20 scale, ranging from *very unpleasant* to *very pleasant*) and “How much do you want to eat this type of food right now?” (rated on a 0 to 20 scale, ranging from *not at all* to *very much*).

### Data Analysis

Analyses were conducted with SPSS 24.0 and R (IBM Corp., 2015; R Core Team, 2013). To test whether the overtraining manipulation caused more habitual responding in the 3-day group versus the 1-day group, the change in responses per second from training to extinction test was the dependent measure in a repeated-measures ANOVA with group (1-day, 3-day) as a between-participants factor and value (devalued, valued) as a within-participant factor. Bayes factors complement frequentist statistics for the critical Group × Value interaction analysis. An identical analysis was performed on the raw performance data from the end of training, using the last three blocks of each block type completed during training. To test whether the devaluation manipulation was successful in decreasing the desirability of the devalued food, we conducted a repeated-measures ANOVA on participants’ ratings of how much they wanted the foods at a given moment. Group (1-day, 3-day) was a between-participants factor and time (predevaluation, postdevaluation) and value (valued, devalued) were within-participant factors.

## Results and Discussion

### Training Phase

**Figure 8 fig8:**
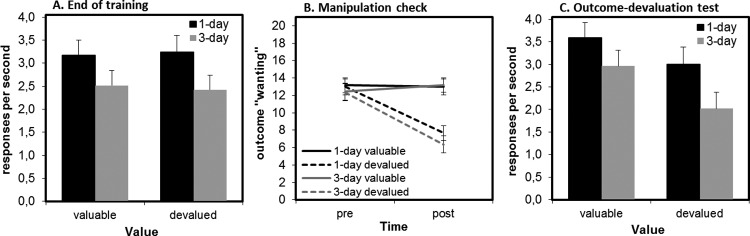
Main results of Experiment 3A. Panel A: End of training. Responses per second for the to-be-devalued outcome and the to-be-valuable outcome at the end of training (averaged across last three blocks of valued and devalued block-types). Response rates are shown separately for the 1-day training group (black bars) and the 3-day training group (gray bars). Panel B: Manipulation check. Food “wanting” ratings on 0 to 20 Likert scales (“How much do you want to eat this type of food right now?”) at pre- and postdevaluation manipulation. Scores are shown separately for the 1-day training group (black lines) and the 3-day training group (gray lines). Dashed lines represent ratings of the devalued food, and solid lines ratings of the valuable food. Panel C: Response rates during the outcome-devaluation test, shown separately for the brief and extended training groups. Error bars represent standard error of the means.

During the final three blocks of training for each block type, there was no main effect of value (to-be devalued, to-be valued) and no interaction between value and group (*F* < 1). There was also no significant main effect of group, *F*(1, 62) = 2.60, *p* = .113 (see Figure 8, Panel A). Participants in the 1-day group made an average of 1,897 responses over the course of their one day of training (range = 73–3,409; *SD* = 965), whereas the 3-day group made an average of 8,642 responses over their 3 days (range = 963–16,624 presses; *SD* = 4,811).[Fig-anchor fig8]

#### Manipulation Check

To investigate whether the selective satiation manipulation was successful, we analyzed self-reported current level of “wanting” the two food items. We found a significant interaction between time (predevaluation, postdevaluation) and value (valued, devalued), *F*(1, 62) = 61.08, *p* < .001. Follow-up tests of simple effects showed that this interaction was driven by significantly lower wanting of the devalued outcome relative to the value outcome postdevaluation, *F*(1, 62) = 56.00, *p* < .001, but not predevaluation (*F* < 1). There was no significant main effect or interactions with group (all *F*s < 1.22, all *p*s > .27), indicating the devaluation was equally effective in both groups (see Figure 8, Panel B). For other ratings results, see the text of the online supplemental material and Table 3A. Data regarding the quantity (weight in grams) of food consumed were missing for 5 participants. Of those remaining (*N* = 59), participants ate on average 40.4 g during the devaluation manipulation (*SD* = 33.2; range = 2.1 g −143.8 g). Importantly, there was no difference between the 1-day and 3-day groups in amount consumed, *t*(57) = .30, *p* = .77.

### Outcome-Devaluation Test

To assess if our training duration manipulation had successfully induced habits in the extensively trained group (3-day) relative to the briefly trained group (1-day), we examined participants’ change in responding from training to extinction (as in Experiments 1A and 1B), therefore controlling for baseline response rates. We found a significant main effect of value on the change in responding from training to test, *F*(1, 62) = 13.61, *p* < .001, indicating that overall participants showed sensitivity to devaluation, reducing their responding for devalued outcomes more than for valued outcomes. There was no main effect of group (*F* < 1) and, most critically, no interaction between group and value (*F* < 1). Bayesian model comparison revealed moderate evidence against including the interaction term (Bayes factor = 3.96).

Despite the nonsignificance of the critical interaction, we nonetheless ran post hoc tests of simple effects to permit a more detailed comparison with the original study by Tricomi and colleagues. In the 1-day group, there was a significant main effect of value, *F*(1, 32) = 9.33, *p* = .005, with a positive change from training to test for the valued outcome (i.e., an increase in responding of .42 responses per s; *SD* = 1.2) and a negative change from training to test for the devalued outcome (i.e., a decrease in responding of .24; *SD* = 1.2). In the 3-day group, there was also a significant main effect of value, *F*(1, 32) = 5.73, *p* = .023. This was driven by a positive change from training to test for the valued outcome (i.e., an increase in responding of .44 responses per s; *SD* = 1.1) and a negative change from training to test for the devalued outcome (i.e., a decrease in responding of .40; *SD* = 1.7). Although speculative, this pattern of increasing responding to the valued stimulus might suggest an increase in response vigor following the break between training and test. Importantly, this did not differ across groups and does not impinge on the critical test for habit formation, which is the comparison of valued and devalued responding in extinction.

### Summary and Conclusions

In contrast to Tricomi et al., (2009), we did not find any evidence for habit induction as a consequence of overtraining. Overall, participants responded more for the valuable than for the devalued outcome, but contrary to our expectation, this did not differ as a function of training duration (1-day vs. 3-day).

## Experiment 3B

What follows is an independent, parallel attempt to replicate the findings from Tricomi et al. (2009), this time carried out in the Netherlands. As in Experiment 3A, we aimed to copy the original procedure as closely as possible. Nonetheless, there were a few minor differences, which are detailed in the following Method section. Furthermore, we included additional measures at the end of the experiment, namely an adapted version of the SOAT and the Self-Report Behavioral Automaticity Index (SRBAI; Gardner, Abraham, Lally, & de Bruijn, 2012). These measures were included to gain more information about the balance between goal-directed and habitual control. The SRBAI is a self-report measure that has been used extensively in health psychology to reveal (experienced) automaticity as a function of behavioral repetition. We hypothesized, therefore, that the 3-day group should score higher on this measure than the 1-day group. The additional SOAT was similar to the test adopted in the original study of Tricomi and colleagues, only, this time, participants were explicitly instructed to just respond for the more desirable outcome out of the two. Therefore, this test resolved any ambiguity that may have arisen in the original test. Furthermore, this time the discriminative stimuli were shown only very briefly, such that participants had to respond fast. Time pressure is thought to favor habitual (over goal-directed) responding, and this feature may therefore render the slips-of-action test more sensitive to habit strength.

## Method

### Participants

Fifty-one healthy participants participated in this experiment (42 females; *M* ± *SD* age 22.71 ± 3.58; range = 18–34 years; *M* ± *SD* BMI 21.97 ± 2.70; range = 18–33). Before entering the study, participants were informed that they should be willing to consume M&Ms and salty popcorn in this study. Participants with a history of eating disorders were excluded. Participants received 35 euros (equivalent to ~$41; or 3.5 student credits) compensation. They were required to fast for 2 hr prior to each experimental session. All participants gave written informed consent and the study conforms with the Ethical Committee of the University of Amsterdam.

### Materials

The task was programmed in Presentation software (Neurobehavioral Systems, Inc., Berkeley, CA, www.neurobs.com). M&Ms and salty popcorn functioned as the reward.

### Procedure

Participants were randomly assigned to a 1-day training group (*N* = 24) and a 3-day training group (*N* = 27). The groups were matched for age, *t*(49) = −1.34, *p* = .19, gender (χ^2^ < .83, *p* = .36), and BMI, *t*(49) = 1.09, *p* = .28.

#### Training

The instrumental training phase took place in the same way as in the previous studies, except for the following differences. First, the fractals had a fixed duration of 20 s, and were each presented 16 times during each session, randomly intermixed with 16 rest blocks of 12 s each. The 1-day group completed one session on 1 day. The 3-day group completed two sessions (with one short break in the middle: “Please press the spacebar when you’re ready to continue with the task”) on each of 3 consecutive days. Therefore, the total number of training blocks were 16 for the 1-day training group and 96 for the 3-day group. At the start of each day, the training instructions were reiterated, as they were in Experiment 3A. No practice trials were provided. Second, prior to the training phase, participants were instructed that they were earning points for snacks to eat while watching a TV series. At the end of each training day, participants were accompanied to a separate “TV room,” in which the lights were dimmed, and they could sit in a comfortable chair. They were told that they had a collection of *Inside Amy Schumer* videos available to choose from and that they would have 10 min to watch these. In the meantime, they could eat the popcorn and M&Ms that they earned during the game (the total number of points earned was divided by eight to calculate the number of grams of each snack reward). Participants were also offered a glass of water. The experimenter left the room. Finally, if participants asked for clarification during the task, the experimenter repeated the complete initial instruction: “You can press the button as often or as little as you like. If you do not want any more Fritos or M&Ms, you don’t have to continue pressing.” This is slightly more explicit than in Experiment 3A and the original study by Tricomi and colleagues (2009), where the clarification was as follows: “You can press the button as often or as little as you like.”

#### Devaluation manipulation

The outcome-devaluation manipulation took place immediately after participants had consumed the snacks that they earned during the game. They remained in the TV room and were told that they were offered an additional amount of one of the snacks as a bonus. Half of the participants was given 160 g of M&Ms and the other half 160 g of popcorn. They were instructed that they could request more, after which the experimenter left the room again. After 10 min the experimenter returned and asked if they still wanted to consume more. If not, participants were returned to the testing room.

#### Outcome-devaluation test

Participants were told that they would play the same game as before. The ensuing extinction test consisted of three 20-s presentations of each of the two fractals in random order and three 12-s resting stimuli that were randomly interspersed. Rewards were not presented during this phase. The gray dot still appeared for every click.

#### SOAT

Participants were instructed that they would perform one final task in which they could earn more points for the snacks by pressing keys upon seeing the fractal stimuli. Each fractal was shown for only 800 ms and was followed by the next fractal after an ITI of 1,000 ms.

This time you have to react fast as the stimuli will quickly disappear from the screen. If you react correctly and in time we won’t provide you with immediate feedback, but you will in fact be earning points. Next to the abstract images, you will sometimes be shown arrows: if these are pointed to the left you should press left, and vice versa if pointed toward the right.

Subsequently, they were asked to indicate for which snack they wished to work still: either for the popcorn or for the M&Ms. They were told to only respond for the preferred snack during this final test phase. The SOAT consisted of nine blocks, during each of which the two fractal stimuli were shown three times, and the arrows each two times, in random order (the corresponding response buttons were no longer shown at the top of the screen). Therefore, the total number of trials with each fractal was 27. After the SOAT, they were seated in the TV room again where they could consume the earned snacks while watching Amy Schumer.

#### Manipulation check

In this experiment, hunger and wanting ratings were given on 0 to 10 scales (instead of 0 to 20 scales, as in Experiment 3A). Furthermore, participants provided these ratings at three time points: at the start of the experiment, immediately after the devaluation manipulation, and at the end of the experiment.

#### Exit measures

In the exit questionnaire participants answered a list of additional questions. This questionnaire included the four SRBAI items (Gardner et al., 2012) that were rephrased to apply to the experimental task: “Upon seeing the fractal images, I pressed automatically; I pressed without thinking; I pressed without having to consciously remember to; I started pressing before I realized I was doing it.” There were several additional items in the questionnaire, which are reported (together with the hunger ratings) in the supplemental text and in Table 3B of the online supplemental material. After completing the questionnaire, participants were weighed and their height was determined, to calculate their BMI.

### Data Analysis

We conducted identical analyses to those reported for Experiment 3A (with SPSS Version 22.0 and R; IBM Corp., 2015; R Core Team, 2013). Additionally, to analyze whether the mean presses for preferred valued and nonpreferred devalued outcomes during the SOAT differed between the 1-day and 3-day training condition, a repeated-measures ANOVA was performed on the mean amount of presses with the within-participant variable value (valued vs. devalued) and between-participants variable group (1-day vs. 3-day training).

## Results and Discussion

**Figure 9 fig9:**
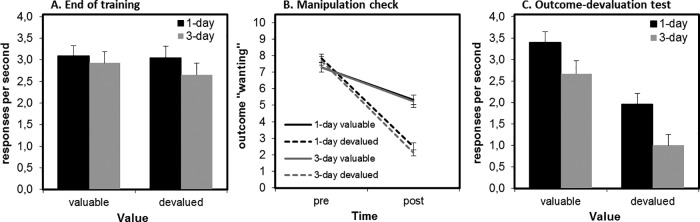
Main results of Experiment 3B. Panel A: End of training. Responses per second for the to-be-devalued outcome and the to-remain-valuable outcome at the end of training (averaged across last three blocks of valued and devalued block-types). Response rates are shown separately for the 1-day training group (black bars) and the 3-day training group (gray bars). Panel B: Manipulation check. Food ‘wanting’ ratings on 0 to 10 Likert scales (“How much do you want to eat this type of food right now?”) pre- and postdevaluation manipulation. Scores are shown separately for the 1-day training group (black lines) and the 3-day training groups (gray lines). Dashed lines represent ratings of the devalued food, and solid lines ratings of the valuable food. Panel C: Response rates during the outcome-devaluation test, shown separately for the 1-day and 3-day training groups. Error bars represent standard error of the means.

Responding during the final three blocks of training is depicted in Figure 9, Panel A. There was no main effect of value or an interaction between value and group (*F*s < 1). There was also no main effect of group (*F* < 1). Participants in the 1-day group made an average of 2,105 responses over the course of their 1 day of training (range = 425–3,734; *SD* = 1,047), whereas the 3-day group made an average of 11,407 responses over their 3 days (range = 725–19,113; *SD* = 5447).[Fig-anchor fig9]

### Manipulation Check

Participants ate on average 50.43 g during the devaluation manipulation (*SD* = 27.42; range = 8–131 g). Importantly, there was no difference between the 1-day and 3-day groups in amount consumed, *t*(49) = .89, *p* = .38. Analysis of self-reported wanting of the food rewards showed a significant interaction between time and value, *F*(1, 47) = 89.45, *p* < .001. Follow-up analyses confirmed that this interaction was driven by significantly reduced wanting of the devalued outcome relative to the valuable outcome postdevaluation, *F*(1, 49) = 56.33, *p* < .001, but not predevaluation, *F*(1, 47) = 2.66, *p* = .11. There was no main effect or interaction with group (all *F*s < 1, *p*s > .67), indicating that the devaluation was equally effective in the two groups (see Figure 9, Panel B).

### Outcome-Devaluation Test

There was a significant main effect of value, *F*(1, 49) = 28.73, *p* < .001, such that participants decreased responding more toward the devalued compared with valued outcomes. As in Experiment 1A, there was no interaction between group and value (*F* < 1). In contrast to our hypothesis, the groups were equally sensitive to devaluation, despite the considerable differences between their training durations (see Figure 9, Panel C). Moreover, Bayesian model comparison revealed moderate evidence against including the interaction term (Bayes factor = 3.71). There was no significant main effect of group, *F*(1, 49) = 2.86, *p* = .097, but the trend was for the 3-day group to reduce their responding overall more than the 1-day group.

As in Experiment 1A, we also ran post hoc tests of simple effects to facilitate the comparison with the original study by Tricomi and colleagues (2009). In the 1-day group, there was a significant main effect of value *F*(1, 23) = 12.55, *p* = .002. These participants had a small positive change from training to test for the valued outcome (i.e., an increase in responding of .31 responses per s; *SD* = 1.4) and a larger negative change from training to test for the devalued outcome (i.e., a decrease in responding of 1.1; *SD* = 1.6). In the 3-day group, there was also a significant main effect of value *F*(1, 26) = 16.43, *p* < .001. This was driven by a small decrease from training to test for the valued outcome (i.e., decrease in responding of .26 responses per s; *SD* = 1.3) and a larger reduction from training to test for the devalued outcome (i.e., a decrease in responding of 1.65; *SD* = 1.6).

### SOAT

This analysis revealed a significant main effect of value, *F*(1, 49) = 109.09, *p* < .001, η^2^ = 0.69, but once again no significant interaction between value and group (*F* < 1). Participants pressed more for valuable outcomes (*M* = 22.45, *SD* = 7.61) than for devalued outcomes (*M* = 2.59, *SD* = 7.78).

### Self-Reported Automaticity

An independent samples *t* test on the overall SRBAI scores showed no difference in mean overall SRBAI score between the 1-day and 3-day training group, *t*(49) = 1.69, *p* = .10, with average scores of 3.51 and 4.31 (*SD*s = 1.49 and 1.86), respectively. Separate *t* tests on the four SRBAI items showed a marginally significant higher score of the 3-day group on the following item: “I pressed without thinking,” *t*(49) = 1.79, *p* = .079, and a significantly higher score on the following item: “I pressed without having to consciously remember to,” *t*(49) = 2.99, *p* = .004. The scores on the other two items did not differ between the groups, *t*s(49) < .99, *p*s > .33.Therefore, the SRBAI provides modest support for a subjective increase in automaticity as a result of prolonged training.

### Summary and Conclusions

We found no evidence for increased habit formation with overtraining using this paradigm. Therefore, Experiment 3B (like Experiment 3A) failed to replicate the previously reported effect of overtraining on outcome devaluation sensitivity (Tricomi et al., 2009).

In Experiment 3B, we used a slightly different satiation procedure than in Experiment 3A and the original study by Tricomi and colleagues (2009), but we found that it was equally effective. In the extinction test, participants in both the 1-day and 3-day groups preferentially responded for the valued relative to the devalued outcomes. Next, we conducted an additional test, the SOAT. This test offered explicit instructions to selectively respond for the still-valuable outcome, thereby resolving any ambiguity that may have affected performance in the first extinction test. At the same time, this test might have been more sensitive to overtraining because it was conducted under time pressure. However, we found that participants in both groups performed at a very high level on this task, making almost no responses for the devalued outcomes. Self-reported automaticity did appear to increase with extensive training, but in the absence of a behavioral effect of extensive training, this finding is of limited scope. Future research that successfully induces habits in overtrained compared with undertrained participants could relate this subjective measure to objective performance.

Other subtle differences between our procedure and that of the original study, which do not appear in the original published article, were revealed in personal communications with E. Tricomi (May 5, 2017). Specifically, (a) in both Experiment 3A and Experiment 3B, participants received the instruction, “If you do not want any more Fritos or M&Ms, you don’t have to continue pressing,” at the beginning of each day of training, not just on Day 1 as in the study of Tricomi and colleagues (2009). Additionally, (b) on rare occasions that a participant asked for clarification regarding whether they should continue pressing post devaluation, whereas Experiment 3A and the original Tricomi et al. article stated “You can press the button as often or as little as you like,” Experiment 3B repeated the entire original instruction: “If you do not want any more Fritos or M&Ms, you don’t have to continue pressing.” Potentially, these additional instructions in Experiments 3A and 3B made participants in the 3-day group more aware of the task requirements than participants in the 3-day group in the original demonstration and may have thus led to a relative preservation of goal-directed control. Conversely, it could be argued that the original demonstration of superior devaluation sensitivity in the 1-day training group in Tricomi’s study was due to the recency of this instruction rather than to the number of S-R repetitions, as this explicit instruction was supplied on the same day as testing in the minimal group, in contrast to the 3-day group that was tested 2 days postinstruction. The authors suspect that these details are minor, but we provide this information nonetheless so that the interested reader may consider these issues in future.

## General Discussion

The five experiments reported here aimed to produce habits in humans via overtraining. Across all studies, we manipulated the duration of instrumental training that participants received, and then implemented an outcome-devaluation procedure, where previously rewarding outcomes were made less valuable. We then tested if instrumental behavior would show sensitivity to this change in outcome value by monitoring subsequent choice behavior in an extinction test. Participants who formed habits should show less sensitivity to devaluation; continuing to automatically respond for outcomes that are no longer of value. In Experiments 1A and 1B, an instrumental avoidance task was used; in Experiment 2, we used an appetitive SOAT; and in Experiment 3, we adopted an appetitive outcome-devaluation task that had previously been used to provide the sole experimental demonstration of habits following an overtraining manipulation in humans (Tricomi et al., 2009). Contrary to our hypotheses, none of these experiments provided evidence for increased habits as a consequence of extensive training in humans. We should point out that we did observe residual responding for devalued outcomes in all experiments, and particularly strongly in Experiments 3A and 3B, which is consistent with the formation of a habit. However, we failed to experimentally manipulate the extent of devalued responding as a function of training duration. Null effects should be interpreted with caution; it remains entirely possible that more extensive training would have revealed habit formation using the paradigms we investigated, or that subtle adjustments to these different task designs would render them more sensitive to overtraining effects on habit formation. However, we believe that these findings show quite clearly that it is not trivial to experimentally induce habits in healthy humans as a function of behavioral repetition and that there currently exists no procedure that can reliably be used to do so.

Three of the tasks (Experiments 1A, 1B, and 2) used in the current series of experiments offered a choice (between a left and a right response), albeit that discriminative stimuli signaled that only one of these was likely to result in reward or the cancellation of an aversive event. One important consideration might be that successful demonstrations of outcome-insensitive habits in animals have employed very simple designs in which rats were trained to perform a single response to obtain a single outcome (Adams, 1982). Kosaki and Dickinson (2010) showed that providing rats with a choice between responses during the learning phase prevents the formation of behavioral autonomy, such that rats remain goal-directed even after extensive training (see also Colwill & Rescorla, 1985). However, the final two studies (Experiments 3A and 3B) could be argued to be similar to the animal studies in this respect, as only one button was made available to participants per block. However, as opposed to the animal studies, training was not blocked. In Experiments 3A and 3B and in the study of Tricomi et al. (2009), right button blocks were randomly intermixed with left button blocks. In contrast, blocked training—as in the animal studies—produces fixed action sequences, which may contribute to the formation of habits as a function of behavioral repetition (Dezfouli, Lingawi, & Balleine, 2014). Further research is required to gain insight into the role of action sequences in habit formation. In any case, a potentially promising approach in future studies could be to design a paradigm that is more similar to the simple setup of the original animal demonstrations, with a single response-outcome contingency. To prevent a conscious, goal-directed strategy from dominating performance, experimenters should probably embed such a simple task within a more complex task or be offered concurrently with another task, distractors, or stressors (Lin, Wood, & Monterosso, 2015). In other words, taxing goal-directed control may be required to reveal the gradual build-up of habits in healthy humans in an experimental testing environment. Relatedly, we cannot eliminate the possibility that this partially explains our failure to replicate the findings of Tricomi and colleagues. Their study was conducted within the noisy and putatively stressful environment of an fMRI scanner, which may have led to accelerated formation of strong habits.

One limitation of the present collection of studies is the nonsystematic nature of the progression from one experiment to the next. Rather than varying one key element, for example, positive versus negative reinforcers, we used tests that varied on numerous key task parameters. Although this does impose limits on what can be concluded by comparing across tests, the choice of paradigms has important strengths. Namely, it enables us to make inferences about prior research that have used these tasks to probe the balance between goal-directed behavior and habits in psychopathology. For example, a previous study found evidence to suggest that OCD patients showed increased habits compared with control participants, but only after extended training (Gillan et al., 2014). One possibility is that both groups may have acquired stronger habitual S-R associations following extended training, but that these were only expressed in the OCD group because these patients have impairments in goal-directed control. In the healthy controls, perhaps intact goal-directed control faculties mean that they can easily override those S-R habits (Gillan & Robbins, 2014). In line with this view, a previous study showed that the failures in devaluation sensitivity in OCD patients on the SOAT correlated with their knowledge of the R-O contingencies and not with knowledge of the S-R contingencies (Gillan et al., 2011). Moreover, a follow-up fMRI study with the avoidance task provided further evidence for the notion that OCD is best characterized in terms of impaired goal-directed control, by relating devaluation-insensitive behavior to hyperactivity in the caudate, a structure previously implicated in goal-directed control (Gillan, Apergis-Schoute, et al., 2015), and finding no differences in habit-forming regions like the putamen. This same reasoning could be applied to other studies that have provided evidence for habit propensity in psychopathologies (e.g., Delorme et al., 2016; de Wit et al., 2011; Ersche et al., 2016; Sjoerds et al., 2013) and following various stress manipulations (Schwabe & Wolf, 2009). Impaired task performance in those studies might reflect reduced goal-directed control, rather than differences in habit strength. It should be noted, however, that Delorme et al. (2016) and Sjoerds et al. (2013) did provide preliminary evidence for involvement of the habitual network in outcome-insensitive responding in patients with Tourette syndrome and alcohol dependence.

One of the key issues with the study of habit formation is that habits and goal-directed control are defined reciprocally when using the outcome devaluation test. Behaviorally, an individual who continues to respond despite changes in outcome value may have an increase in habit or a decrease in goal-directed control. Neuroimaging and lesion work in rodents and humans have contributed immensely to this literature, showing what behavior cannot - that distinct brain systems contribute to habits and goal-directed control, and that each system contributes (more or less) independently to one’s likelihood to persist in responding during an outcome devaluation test. New computational approaches offer an alternative means to obtain separate (albeit indirect) assessments of these mechanisms, for example using the two-step sequential decision-making task (Daw, Gershman, Seymour, Dayan, & Dolan, 2011). This approach dissociates model-based and model-free learning algorithms, which have been theorized to reflect goal-directed and habitual strategies. This theory was put to the test recently in two studies (Friedel et al., 2014; Gillan, Otto, et al., 2015), which related model-based and model-free estimates to performance on a simple outcome-devaluation test. Both studies found that model-based learning was a positive predictor of flexible adjustments based on a change in outcome value (i.e., goal-directedness), whereas model-free learning was unrelated to performance on the outcome-devaluation task. Relatedly, a recent study found that performance on the SOAT showed a moderately positive correlation with model-based learning on the two-step task, but once again, bore no relation with model-free learning (Sjoerds et al., 2016). Taken together, these studies are consistent with the notion that model-based reinforcement learning is a good approximation of goal-directed control defined in terms of performance on outcome-devaluation tasks. However, it appears that the model-free reinforcement learning algorithm, much like our overtraining manipulations, is not sensitive to habit strength in humans.

Interestingly, neuroimaging has provided indirect support for the notion of competing goal-directed and habitual systems using outcome-devaluation paradigms (de Wit et al., 2009; de Wit, Watson, et al., 2012; Liljeholm, Molloy, & O’Doherty, 2012; Tricomi et al., 2009; Valentin et al., 2007), suggesting that it should be possible in principle to capture these processes behaviorally. Furthermore, alternative paradigms have been developed to dissociate habitual from goal-directed performance, albeit not as a function of behavioral repetition. Liljeholm et al. (2012) and colleagues manipulated instrumental task contingencies with the aim of encouraging either goal-directed responding based on consistent response-outcome pairings or habitual responding based on S-R pairings. In the S-R condition, participants were subsequently less sensitive to outcome devaluation. In another series of studies, the Pavlovian-instrumental transfer (PIT) paradigm has been used to study stimulus-driven behavior (Cartoni, Balleine, & Baldassarre, 2016). For example, when participants were offered a choice between responding on a left key for popcorn and on a right key for M&Ms, a Pavlovian stimulus that was previously associated with popcorn biased responding toward the left key (Watson et al., 2014). Interestingly, this outcome-specific PIT effect appears to be insensitive to outcome devaluation, even after minimal training, both in animals (Holland, 2004; Rescorla, 1994) and in humans (Hogarth & Chase, 2011; van Steenbergen, Watson, Wiers, Hommel, & de Wit, 2017; Watson et al., 2014). In contrast, when the same participants were tested in the absence of the Pavlovian cues, their performance was sensitive to devaluation. Therefore, this experimental paradigm may offer an alternative approach to behaviorally dissociating stimulus-driven outcome-insensitive responding from goal-directed action (Corbit & Janak, 2016). The congruence paradigm is another procedure that aims to dissociate the contributions of the goal-directed and habitual systems during learning, by rendering action-outcome learning disadvantageous in so-called incongruent discriminations (e.g., de Wit et al., 2009; de Wit, Watson, et al., 2012).

Diary investigations present an alternative approach to studying habits in humans in the context of everyday behaviors. These studies provide evidence that self-reported behavioral intentions are weak predictors of behavior that has been repeated extensively (Quellette & Wood, 1998). For example, self-reported habit strength in terms of the experience of automaticity and frequency of past performance moderates the strength of the relation between intentions and actual behavior (Gardner, de Bruijn, & Lally, 2011). This finding was borne out in an elegant field experiment, where a strong self-reported habit to eat popcorn in the cinema was found to predict consumption of stale popcorn in the context of the cinema, but not in an alternative context (a meeting room; Neal, Wood, Wu, & Kurlander, 2011). However, as valuable as these self-report associations are, it remains an issue that there are limits to how well people can reflect upon their (habitual) behavior. As such, carefully controlled experimental research remains crucial for our understanding of the underlying mechanisms, as well as for the investigation of the neural basis and clinical relevance of habits (de Wit, 2017). We need paradigms that allow us to experimentally manipulate habit expression in order to causally investigate how habits shape our behaviors, beliefs, and intentions.

In conclusion, in this article we presented five attempts to demonstrate a shift in the balance between goal-directed and habitual control as a consequence of extended behavioral repetition. Our findings are consistent with the view that current outcome-devaluation tasks tap predominantly into goal-directed control. This might be because overtrained habits play a moderate role in the outcome sensitivity of human action control or because our training durations are insufficient (or insufficiently spread out over separate days) to instill sufficiently strong habits. To the latter point, one diary study suggests that it takes people on average 66 days (between 18 and 254) to form a new habit (Lally, Van Jaarsveld, Potts, & Wardle, 2010). Our hope is that the presented studies will inspire more fruitful attempts in future, or critical reassessment of our current theoretical framework, or indeed both.

### Context Paragraph

The authors have considerable experience with experimentally investigating instrumental behavior in humans, and more specifically the balance between goal-directed and habitual action control. They both developed tasks that are used by research groups around the world to investigate the neural basis thereof, as well as the clinical relevance. However, they have also for some time been aware of the limitations of these paradigms, in terms of revealing the specific contributions of the two underlying systems—habitual and goal-directed (as opposed to simply discussing the balance between them). For this reason, they have independently tried to establish a paradigm with which to study a gradual shift from goal-directed to habitual control with overtraining. However, this translational step from animal to human research has proven a challenging one. When we found out that we had both been struggling to find evidence for overtrained habit independently, we decided to open the file drawer and publish our experiments together. We believe it is important to bring these findings to the attention of our colleagues, because based on this collection of studies, we believe that the relationship between overtraining and habits in humans has not been satisfactorily demonstrated in a manner akin to the original animal demonstrations. Our hope is that recognizing that it is nontrivial to instill habits in humans using this kind of procedure will inspire the development of new paradigms, theories and more critical investigation regarding this issue.

## Supplementary Material

10.1037/xge0000402.supp

## Figures and Tables

**Table 1 tbl1:** Summary of Study Designs

Exp	Sample size	Schedule	*N* training trials per stimulus	*N* test trials per stimulus	Outcome
1A	36,38	FR-1	2 vs. 33	3	Shock (−)
1B	24,24,24	FR-1	4 vs. 48 vs. 96 in 2 days	8	Noise (−)
2	43	FR-1	32 vs. 160 in 3 days	8	Points (+)
3A	33,31	VI-10	12 vs. 72 in 3 days	3	Food (+)
3B	24,27	VI-10	16 vs. 96 in 3 days	3	Food (+)
*Note*. Exp = experiment; FR = fixed ratio; VI = variable interval; + = appetitive outcome; − = aversive outcome.					

## References

[c1] Adams C. D. (1982). Variations in the sensitivity of instrumental responding to reinforcer devaluation. The Quarterly Journal of Experimental Psychology, 34, 77–98. 10.1080/14640748208400878

[c2] Adams C. D., & Dickinson A. (1981). Instrumental responding following reinforcer devaluation. The Quarterly Journal of Experimental Psychology, 33, 109–121. 10.1080/14640748108400816

[c3] Alvares G. A., Balleine B. W., Whittle L., & Guastella A. J. (2016). Reduced goal-directed action control in autism spectrum disorder. Autism Research, 9, 1285–1293. 10.1002/aur.1613 26999793

[c4] Balleine B. W., & O’Doherty J. P. (2010). Human and rodent homologies in action control: Corticostriatal determinants of goal-directed and habitual action. Neuropsychopharmacology, 35, 48–69. 10.1038/npp.2009.131 19776734 PMC3055420

[c5] Cartoni E., Balleine B., & Baldassarre G. (2016). Appetitive Pavlovian-instrumental transfer: A review. Neuroscience and Biobehavioral Reviews, 71, 829–848. 10.1016/j.neubiorev.2016.09.020 27693227

[c6] Carver C. S., & White T. L. (1994). Behavioral inhibition, behavioral activation, and affective responses to impending reward and punishment: The BIS/BAS Scales. Journal of Personality and Social Psychology, 67, 319–333. 10.1037/0022-3514.67.2.319

[c7] Colwill R. M., & Rescorla R. A. (1985). Instrumental responding remains sensitive to reinforcer devaluation after extensive training. Journal of Experimental Psychology: Animal Behavior Processes, 11, 520–536. 10.1037/0097-7403.11.4.520 2303793

[c8] Corbit L. H., & Janak P. H. (2016). Habitual alcohol seeking: Neural bases and possible relations to alcohol use disorders. Alcoholism, Clinical and Experimental Research, 40, 1380–1389. 10.1111/acer.13094 PMC5600324 27223341

[c9] Daw N. D., Gershman S. J., Seymour B., Dayan P., & Dolan R. J. (2011). Model-based influences on humans’ choices and striatal prediction errors. Neuron, 69, 1204–1215. 10.1016/j.neuron.2011.02.027 21435563 PMC3077926

[c10] Delorme C., Salvador A., Valabrègue R., Roze E., Palminteri S., Vidailhet M., . . . Worbe Y. (2016). Enhanced habit formation in Gilles de la Tourette syndrome. Brain, 139(2), 605–615. 10.1093/brain/awv307 26490329

[c11] de Wit S. (2017). Control of behaviour by competing learning systems In Egner T. (Ed.), The Wiley handbook of cognitive control (pp. 190–206). Chicester, West Sussex: John Wiley & Sons 10.1002/9781118920497.ch11

[c12] de Wit S., Barker R. A., Dickinson A. D., & Cools R. (2011). Habitual versus goal-directed action control in Parkinson disease. Journal of Cognitive Neuroscience, 23, 1218–1229. 10.1162/jocn.2010.21514 20429859

[c13] de Wit S., Corlett P. R., Aitken M. R., Dickinson A., & Fletcher P. C. (2009). Differential engagement of the ventromedial prefrontal cortex by goal-directed and habitual behavior toward food pictures in humans. The Journal of Neuroscience, 29, 11330–11338. 10.1523/JNEUROSCI.1639-09.2009 19741139 PMC3443853

[c14] de Wit S., & Dickinson A. (2009). Associative theories of goal-directed behaviour: A case for animal-human translational models. Psychological Research, 73, 463–476. 10.1007/s00426-009-0230-6 19350272 PMC2694930

[c15] de Wit S., Niry D., Wariyar R., Aitken M. R., & Dickinson A. (2007). Stimulus–outcome interactions during instrumental discrimination learning by rats and humans. Journal of Experimental Psychology: Animal Behavior Processes, 33, 1–11. 10.1037/0097-7403.33.1.1 17227190

[c16] de Wit S., Ridderinkhof K. R., Fletcher P. C., & Dickinson A. (2013). Resolution of outcome-induced response conflict by humans after extended training. Psychological Research, 77, 780–793. 10.1007/s00426-012-0467-3 23192433

[c17] de Wit S., Standing H. R., Devito E. E., Robinson O. J., Ridderinkhof K. R., Robbins T. W., & Sahakian B. J. (2012). Reliance on habits at the expense of goal-directed control following dopamine precursor depletion. Psychopharmacology, 219, 621–631. 10.1007/s00213-011-2563-2 22134475 PMC3249188

[c18] de Wit S., van de Vijver I., & Ridderinkhof K. R. (2014). Impaired acquisition of goal-directed action in healthy aging. Cognitive, Affective & Behavioral Neuroscience, 14, 647–658. 10.3758/s13415-014-0288-5 24796599

[c19] de Wit S., Watson P., Harsay H. A., Cohen M. X., van de Vijver I., & Ridderinkhof K. R. (2012). Corticostriatal connectivity underlies individual differences in the balance between habitual and goal-directed action control. The Journal of Neuroscience, 32, 12066–12075. 10.1523/JNEUROSCI.1088-12.2012 22933790 PMC6621537

[c20] Dezfouli A., Lingawi N. W., & Balleine B. W. (2014). Habits as action sequences: Hierarchical action control and changes in outcome value. Philosophical Transactions of the Royal Society of London. Series B, Biological Sciences. Advance online publication 10.1098/rstb.2013.0482 PMC4186235 25267824

[c21] Dickinson A., Balleine B. W., Watt A., Gonzalez F., & Boakes R. A. (1995). Motivational control after extended instrumental training. Animal Learning & Behavior, 23, 197–206. 10.3758/BF03199935

[c22] Dickinson A., Nicholas D. J., & Adams C. D. (1983). The effect of instrumental contingency on susceptibility to reinforcer devaluation. Quarterly Journal of Experimental Psychology, 35, 249–263. 10.1080/14640748308400909

[c23] Dietrich A., de Wit S., & Horstmann A. (2016). General habit propensity relates to the sensation seeking subdomain of impulsivity but not obesity. Frontiers in Behavioral Neuroscience, 10, 213. 27877117 10.3389/fnbeh.2016.00213 PMC5099246

[c24] Ersche K. D., Gillan C. M., Jones P. S., Williams G. B., Ward L. H. E., Luijten M., . . . Robbins T. W. (2016). Carrots and sticks fail to change behavior in cocaine addiction. Science, 352, 1468–1471. 10.1126/science.aaf3700 27313048 PMC5144994

[c25] Friedel E., Koch S. P., Wendt J., Heinz A., Deserno L., & Schlagenhauf F. (2014, 8). Devaluation and sequential decisions: Linking goal-directed and model-based behavior. Frontiers in Human Neuroscience, 8, 587. 25136310 10.3389/fnhum.2014.00587 PMC4120761

[c26] Gardner B., Abraham C., Lally P., & de Bruijn G. J. (2012). Towards parsimony in habit measurement: Testing the convergent and predictive validity of an automaticity subscale of the Self-Report Habit Index. The International Journal of Behavioral Nutrition and Physical Activity, 9, 102 10.1186/1479-5868-9-102 22935297 PMC3552971

[c27] Gardner B., de Bruijn G. J., & Lally P. (2011). A systematic review and meta-analysis of applications of the Self-Report Habit Index to nutrition and physical activity behaviours. Annals of Behavioral Medicine, 42, 174–187. 10.1007/s12160-011-9282-0 21626256

[c28] Garner D. M., Olmstead M. P., Bohr Y., & Garfinkel P. (1982). The eating attitudes test: Psychometric features and clinical correlates. Psychological Medicine, 12, 871–878. 10.1017/S0033291700049163 6961471

[c29] Geurts H. M., & de Wit S. (2014). Goal-directed action control in children with autism spectrum disorders. Autism, 18, 409–418. 10.1177/1362361313477919 24072663

[c30] Gillan C. M., Apergis-Schoute A. M., Morein-Zamir S., Urcelay G. P., Sule A., Fineberg N. A., . . . Robbins T. W. (2015). Functional neuroimaging of avoidance habits in obsessive-compulsive disorder. The American Journal of Psychiatry, 172, 284–293. 10.1176/appi.ajp.2014.14040525 25526600 PMC4910868

[c31] Gillan C. M., Morein-Zamir S., Urcelay G. P., Sule A., Voon V., Apergis-Schoute A. M., . . . Robbins T. W. (2014). Enhanced avoidance habits in obsessive-compulsive disorder. Biological Psychiatry, 75, 631–638. 10.1016/j.biopsych.2013.02.002 23510580 PMC3988923

[c32] Gillan C. M., Otto A. R., Phelps E. A., & Daw N. D. (2015). Model-based learning protects against forming habits. Cognitive, Affective & Behavioral Neuroscience, 15, 523–536. 10.3758/s13415-015-0347-6 PMC4526597 25801925

[c33] Gillan C. M., Papmeyer M., Morein-Zamir S., Sahakian B. J., Fineberg N. A., Robbins T. W., & de Wit S. (2011). Disruption in the balance between goal-directed behavior and habit learning in obsessive-compulsive disorder. The American Journal of Psychiatry, 168, 718–726. 10.1176/appi.ajp.2011.10071062 21572165 PMC3533260

[c34] Gillan C. M., & Robbins T. W. (2014). Goal-directed learning and obsessive-compulsive disorder. Philosophical Transactions of the Royal Society of London. Series B, Biological Sciences, 369, 20130475 10.1098/rstb.2013.0475 25267818 PMC4186229

[c35] Godier L. R., de Wit S., Pinto A., Steinglass J. E., Greene A. L., Scaife J., . . . Park R. J. (2016). An investigation of habit learning in Anorexia Nervosa. Psychiatry Research, 244, 214–222. 10.1016/j.psychres.2016.07.051 27497292 PMC5718042

[c36] Heyes C., & Dickinson A. (1990). The intentionality of animal action. Mind & Language, 5, 87–103. 10.1111/j.1468-0017.1990.tb00154.x

[c37] Hogarth L., & Chase H. W. (2011). Parallel goal-directed and habitual control of human drug-seeking: Implications for dependence vulnerability. Journal of Experimental Psychology: Animal Behavior Processes, 37, 261–276. 10.1037/a0022913 21500933

[c38] Holland P. C. (2004). Relations between Pavlovian-instrumental transfer and reinforcer devaluation. Journal of Experimental Psychology: Animal Behavior Processes, 30, 104–117. 10.1037/0097-7403.30.2.104 15078120

[c39] Horstmann A., Dietrich A., Mathar D., Pössel M., Villringer A., & Neumann J. (2015). Slave to habit? Obesity is associated with decreased behavioural sensitivity to reward devaluation. Appetite, 87, 175–183. 10.1016/j.appet.2014.12.212 25543077

[c71] IBM Corp. (2015). IBM SPSS Statistics for Windows, Version 24.0 [Computer software]. Armonk, NY: Author.

[c40] Kosaki Y., & Dickinson A. (2010). Choice and contingency in the development of behavioral autonomy during instrumental conditioning. Journal of Experimental Psychology: Animal Behavior Processes, 36, 334–342. 10.1037/a0016887 20658864

[c41] Kruschke J. K. (2013). Bayesian estimation supersedes the *t* test. Journal of Experimental Psychology: General, 142, 573–603. 10.1037/a0029146 22774788

[c42] Lally P., Van Jaarsveld C. H. M., Potts H. W. W., & Wardle J. (2010). How are habits formed: Modelling habit formation in the real world. European Journal of Social Psychology, 40, 998–1009. 10.1002/ejsp.674

[c43] Liljeholm M., Molloy C. J., & O’Doherty J. P. (2012). Dissociable brain systems mediate vicarious learning of stimulus-response and action-outcome contingencies. The Journal of Neuroscience, 32, 9878–9886. 10.1523/JNEUROSCI.0548-12.2012 22815503 PMC3428877

[c72] Lin P. Y., Wood W., & Monterosso J. (2016). Healthy eating habits protect against temptations. Appetite, 103, 432–440. 10.1016/j.appet.2015.11.011 26585633

[c45] Morris R. W., Quail S., Griffiths K. R., Green M. J., & Balleine B. W. (2015). Corticostriatal control of goal-directed action is impaired in schizophrenia. Biological Psychiatry, 77, 187–195. 10.1016/j.biopsych.2014.06.005 25062683

[c46] Neal D. T., Wood W., Wu M., & Kurlander D. (2011). The pull of the past: When do habits persist despite conflict with motives? Personality and Social Psychology Bulletin, 37, 1428–1437. 10.1177/0146167211419863 21859902

[c73] Psychology Software Tools, Inc. (2006). E-Prime 1.2. Retrieved from http://www.pstnet.com

[c47] Quellette J. A., & Wood W. (1998). Habit and intention in everyday life: The multiple processes by which past behavior predicts future behavior. Psychological Bulletin, 124, 54–74. 10.1037/0033-2909.124.1.54

[c74] R Core Team (2013). R: A language and environment for statistical computing [Computer software]. Vienna, Austria: R Foundation for Statistical Computing Retrieved from http://www.R-project.org/

[c48] Rescorla R. A. (1994). Transfer of instrumental control mediated by a devalued outcome. Animal Learning & Behavior, 22, 27–33. 10.3758/BF03199953

[c75] Rickham P. P. (1964). Human experimentation: Code of ethics of the world medical association. Declaration of Helsinki. British Medical Journal, 2, 177 10.1136/bmj.2.5402.177 14150898 PMC1816102

[c49] Schwabe L., & Wolf O. T. (2009). Stress prompts habit behavior in humans. The Journal of Neuroscience, 29, 7191–7198. 10.1523/JNEUROSCI.0979-09.2009 19494141 PMC6666491

[c50] Sjoerds Z., de Wit S., van den Brink W., Robbins T. W., Beekman A. T., Penninx B. W., & Veltman D. J. (2013). Behavioral and neuroimaging evidence for overreliance on habit learning in alcohol-dependent patients. Translational Psychiatry, 3(12), e337 10.1038/tp.2013.107 24346135 PMC4030326

[c51] Sjoerds Z., Dietrich A., Deserno L., de Wit S., Villringer A., Heinze H. J., . . . Horstmann A. (2016). Slips of action and sequential decisions: A cross-validation study of tasks assessing habitual and goal-directed action control. Frontiers in Behavioral Neuroscience, 10, 234 10.3389/fnbeh.2016.00234 28066200 PMC5167743

[c52] Snorrason I., Lee H. J., de Wit S., & Woods D. W. (2016). Are nonclinical obsessive-compulsive symptoms associated with bias toward habits? Psychiatry Research, 241, 221–223. 10.1016/j.psychres.2016.04.067 27183107

[c53] Tricomi E., Balleine B. W., & O’Doherty J. P. (2009). A specific role for posterior dorsolateral striatum in human habit learning. The European Journal of Neuroscience, 29, 2225–2232. 10.1111/j.1460-9568.2009.06796.x 19490086 PMC2758609

[c54] Valentin V. V., Dickinson A., & O’Doherty J. P. (2007). Determining the neural substrates of goal-directed learning in the human brain. The Journal of Neuroscience, 27, 4019–4026. 10.1523/JNEUROSCI.0564-07.2007 17428979 PMC6672546

[c55] van Steenbergen H., Watson P., Wiers R. W., Hommel B., & de Wit S. (2017). Dissociable corticostriatal circuits underlie goal-directed vs. cue-elicited habitual food seeking after satiation: Evidence from a multimodal MRI study. The European Journal of Neuroscience, 46, 1815–1827. 10.1111/ejn.13586 28444823

[c78] Verhoeven A., Kindt M., Zomer C., & de Wit S. (2018). An experimental investigation of breaking learnt habits with verbal implementation intentions. Acta Psychologica, 184, 124–136. 10.1016/j.actpsy.2017.05.008 28552168

[c57] Watson P., Wiers R. W., Hommel B., & de Wit S. (2014). Working for food you don’t desire. Cues interfere with goal-directed food-seeking. Appetite, 79, 139–148. 10.1016/j.appet.2014.04.005 24743030

[c58] Watson P., Wiers R. J., Hommel B., Gerdes V. E. A., & de Wit S. (2017). Stimulus control over action for food in obese versus healthy-weight individuals. Frontiers in Psychology—Eating Behaviors, 8, 580 10.3389/fpsyg.2017.00580 PMC5389979 28450844

[c59] Worbe Y., Savulich G., de Wit S., Fernandez-Egea E., & Robbins T. W. (2015). Tryptophan depletion promotes habitual over goal-directed control of appetitive responding in humans. The International Journal of Neuropsychopharmacology, 18(10), pyv013 10.1093/ijnp/pyv013 25663044 PMC4648160

