# Supplementary material for: Shifting the Balance Between Goals and Habits: Five Failures in Experimental Habit Induction
Source: J Exp Psychol Gen. 2018 Jul;147(7):1043–65. doi: 10.1037/xge0000402 (PMC6033090; doi:10.1037/xge0000402)
Supplement: Supplementary file 1 [file zfr999183041so1.zip › zfr999183041so1.docx]

**Supplemental Materials**

**Shifting the balance between goals and habits: five failures in experimental habit induction**

**By S. de Wit et al., 2018, *Journal of Experimental Psychology: General***

**http://dx.doi.org/10.1037/xge0000402**

*Experiment 1A*

*Skin Conductance Responses*

*Method*

Skin conductance responses (SCRs) were measured using a Biopac system operating Acqknowledge 4.1 (MP36R, Biopac Systems Inc, CA, USA), recording at 1,000 samples per second. SCR was recorded from two Ag-AgCl electrodes, on the distal phalanges on the index and middle fingers of the participants’ non-preferred hand. An isotonic recording gel (Biopac GEL101) was used as the electrolyte. Five minutes were allowed for the gel to absorb before recording. For analysis, SCRs were subject to a threshold of 0.02 umho, and SCRs of less than 5% of the max were excluded. SCRs were identified within 0.5 and 4.5 seconds from the time of stimulus onset and square root transformed prior to analysis. Due to a recording error, skin conductance data from two subjects, one from the 1-Day Brief group and one from the 1-Day Extended group, could not be analyzed.

*Results*

In the outcome devaluation (extinction) test, we found a significant main effect of value, *F*(1,70) = 6.35, *p* =.014, such that SCRs were stronger for the valued (*M* = .63, *SD* =.53) relative to devalued stimulus (*M* = .46, *SD* = .48). Therefore, subjects’ conditioned fear responses (much like their behaviour) reflected the current threat-value of the stimuli during the test stage. This effect was similar across groups; there was no interaction with group *F*(1,70) = 1.63, *p* = .21 and no main effect of group, *F* < 1.

At the end of training (last 2 trials), there was no difference in SCRs between the to-be devalued (*M* =.60, *SD* =.50) and to-be valued stimuli (*M* = .49, *SD* = .43), *F*(1,70) = 2.52, *p* =.12. There was no interaction between group and value, *F*(1,70) = 1.06, *p* =.31 and no main effect of group, *F* < 1.


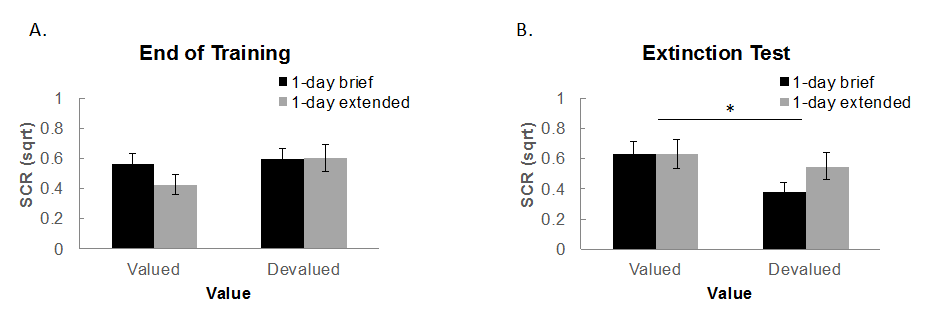


Supplemental Figure 1A. *Skin Conductance Data for Exp 1A. SCR data (square root transformed) at the end of Training (panel A) and in the extinction test (panel B). Error bars reflect standard error of the mean.*

*Exploratory gender analysis*

We repeated our critical repeated measures ANOVA with gender instead of training duration group for the purpose of an exploratory analysis of potential gender effects. We found a significant gender x value interaction, *F*(1,72)=4.498, *p*=.037. However, tests of simple effects showed no gender differences in responding to either valued or devalued outcomes. The interaction was driven by the observation that Females tended to reduce responding to the devalued stimulus to a lesser extent than males, *F*(1,72)=2.685, *p*=.11, while showing a tendency to have a greater reduction in responding to the values stimulus compared to males, F(1,72)=1.92, p=.17. There was no main effect Gender on responding overall in the extinction test, *F*<1

***Experiment 1B***

*Skin Conductance Responses*

Skin conductance data were also recorded for all subjects in this study; however, a subset of these data could not be recovered and so analyses are not presented.

*Results*

Noises were judged as less unpleasant at the post-experiment (*M* = 66.5, *SD* = 16.5) compared to pre-experiment (*M* = 70.2, *SD* = 14.8), evidenced by a main effect of Time on discomfort/irritation ratings, *F*(1,69) = 5.67, *p* = .020. There was no interaction with group, *F*(2,69) = 1.74, *p* = .18, but there was a non-significant trend towards a main effect of group, *F*(1,69) = 2.59, *p* = .082. This was driven by higher overall unpleasantness ratings in the 2-Day group (*M* = 73.6, *SD* = 13.7), compared to the 1-Day Extended group (*M* = 66.3, *SD* = 9.7), *F*(1,46) = 4.55, *p* = .038 and the 1-Day Brief group (*M* = 65.1, *SD* = 17.3), *F*(1,46) = 3.50, *p* = .068. There were no differences between the 1-Day Brief group and the 1-Day Extended group, *F* < 1.

*Exploratory gender analysis*

We did not find evidence for a gender x value interaction, *F*<1 nor was there a main effect of gender on responding overall in the extinction test , *F*(1,70)=1.11, *p*=.296.

***Experiment 2***

*Intention formulation*

At the start of the experiment, participants were asked to write down the labels that they thought best described each of the different pictorial stimuli that were used in the SOAT (e.g. ‘triangle’). These labels were used later in (i) ‘implementation intentions’ or (ii) ‘goal intentions’ that subjects were asked to formulate during the devaluation screens. Half of the participants were assigned to the goal intention condition and the other half to the implementation intention condition. The goal intention condition involved naming out loud the still-valuable outcomes for which they would press. The implementation intention involved naming the stimuli to which they should press using an ‘if-then’ sentence. They were carefully instructed as to the correct sentence structure; for the goal intentions: *“I will still press for X and Y”*; and for the implementation intentions: *“If I see X or Y, then I will still press”*. They had to formulate these intentions themselves on the basis of the visual display of still-valuable outcomes and devalued outcomes (on the devaluation screens at the start of each block). Our expectation was that implementation intentions would lead to more goal-directed performance during the test phase relative to goal intentions. This hypothesis was not supported in the present study (there was no Value X Intention interaction, *F* < 1, nor a Value X Intention X Training duration interaction, *F* < 1). This finding is not in line with two other experiments with the same paradigm in which implementations were shown to be effective (Verhoeven, Kindt, Zomer, & de Wit, 2017). A difference between the studies is that here we did not train participants to repeat the intentions for the duration of the devaluation instruction screens. Consequently, participants in the goal intention condition had ample time to spontaneously formulate implementation intentions. This may have masked the effectiveness of the explicit implementation intention instruction. Because this manipulation was not directly relevant to the present research question, the reported analyses leave out this variable (but, importantly, including it in the analyses did not change the pattern of results). Furthermore, participants filled out the BIS/BAS at the end of the experiment (Carver & White, 1994), but these data are also omitted as this is not directly related to the overtraining hypothesis.

*Exploratory gender analysis*

There was no significant gender x value interaction, *F*(1,41)=2.20, p=.15, nor was there a main effect of gender on responding overall in the extinction test, *F*<1.

***Experiment 3A***

*Full training instructions*

Practice and full instructions for experiment, which were provided at the beginning of each day: “In this game, you will press buttons to earn food that you can eat when you are finished.  Place the fingers of your dominant hand over the r, t, y, and u keys. You will see a fractal appear on the screen. You will also see boxes on the screen corresponding to the keyboard keys. One will be lit. When you press the yellow key, you will see a grey circle appear, this means that your response has been registered. We will practice working towards earning an apple. Try pressing right now” [We rewarded every subject on their 6^th^ press with a picture of an apple.] “If you see a food picture, it means that you have earned that type of food. You can press the button as often or as little as you like. We will now do a longer practice. Feel free to ask the experimenter if you do not understand something.” [Subjects then played for 20 seconds, receiving apple rewards in accordance with the same VI-10 schedule of the main game]. “Well done, that concludes the practice. In the real game, you will be playing for Fritos….” [showed picture of frito icon]. “and M&Ms…” [showed picture of M&M icon]. “If you do not want any more Fritos or M&Ms, you do not have to continue pressing; otherwise you should try to earn as much reward as possible. You should pay attention to which fractal pictures go with which responses and rewards, because we will test you on this at the end of the experiment. At the end of the experiment today, you will eat the food you have earned. You cannot take any food home with you. The task will contain rest periods as well as respond periods. In the rest state, please relax and do not press any keys.  You cannot earn any rewards during the rest state. We are ready to begin. Get Ready! Press any key to begin the experiment”

**Miscellaneous**

*Hunger ratings.* Hunger ratings decreased from pre- to post-devaluation, *F*(1,62) = 64.43, *p* < .001 (Supplemental Table 3A). There was no main effect of Group and no interaction between Group and Time, *Fs* < 1.

*Additional measures.* The goal of this study was to test if experimental habit induction affected subjects’ appraisals of food rewards. We aimed to test if habits could shape beliefs and values *post hoc*, as a means of explaining otherwise irrational habitual responding. To test this, we included measures that assess value from the implicit to the explicit. Along that spectrum, we used (i) an Implicit Attitudes Test, designed to test implicit preferences for M&Ms and Fritos and (ii) a willingness-to-pay task, which measured how much subjects would pay for M&Ms and Fritos (and other control food stimuli) in an auction-style scenario. We asked subjects to rate how much they usually like the two food outcomes twice, pre and post-devaluation. Finally, at the end of the experiment, (iii) we also asked subjects to rate several statements on a Likert scale. On a scale from ‘not at all’ to ‘very much so’, subjects rated the following statements:

- *I was trying to earn snacks because I wanted to eat them afterwards*
- *I was just trying to do well at the task, I did not care about the snacks*
- *I was not really thinking about what I was doing when I was pressing the buttons*
- *Even though I ate a lot of my bonus prize food, I still wanted to earn more*
- *I did not know if I should keep trying to earn the food that I had eaten a lot of*
- *Pressing the buttons took effort*

We hypothesised that subjects in the 3-Day group would increase their valuation of the devalued food post-hoc, as a means of explaining their habitual behaviour in the outcome devaluation test. We were interested to investigate the level of awareness at which this change would occur, from the most implicit (IAT) to most explicit (cognitive ratings). Unfortunately, this analysis was predicated on the assumption that habits would be differentially induced in the 3-Day group and as this was not supported by the data, these analyses were not be performed.

For exploratory purposes, we nonetheless report the results of Spearman’s Rho correlations with these measures and individual differences in devaluation sensitivity (Valued – Devalued responding in the extinction test) observed across the entire sample. We computed 6 correlations with the cognitive questions above. We thus adjusted the significance threshold to *p*<.008 in line with Bonferroni correction. There was only significant correlation between scores on the item *“even though I ate a lot of my bonus food, I still wanted more”* and task performance, *r* = -.338, *p* = .006. Therefore, the more people endorsed this item, the more they continued to press for the devalued food.

Additionally, we included a novel typing flexibility task, which we hypothesised would relate to individual differences in goal-directed control over action (independent of the habit induction manipulation). This entailed first copying a set of words printed on screen under time pressure, and then completing the same task, using a keyboard where the Y and Z keys were reversed. The difference in the number of completed words across conditions was a measure of inflexibility, which we predicted would correlate with devaluation sensitivity (Valued – Devalued Presses in Extinction) in the devaluation test. We found no correlation between devaluation sensitivity and typing interference, *r* = .019, *p* = .88, and as such, the hypothesis was not supported.

Supplemental Table 3A. *Descriptive statistics for self-report measures by group (1-Day, 3-Day) for Experiment 3A. Pre = pre-devaluation (selective satiety) manipulation; Post = post-devaluation*

All ratings were completed on Likert scales from 1-20

|  | **1-Day**  **(*N* = 33)** | | **3-Day**  **(*N* = 31)** | |  |
| --- | --- | --- | --- | --- | --- |
|  | ***M*** | ***SD*** | ***M*** | ***SD*** | **t-test (2-tailed)** |
| **‘How hungry are you right now?’** | |  |  |  |  |
| Hunger (pre) | 16.21 | 3.29 | 15.16 | 3.27 | *t*(62) = 1.30, *p =* .20 |
| Hunger (post) | 10.67 | 4.04 | 10.52 | 4.31 | *t*(62) = 0.14, *p =* .89 |
| **‘How pleasant do you usually find this type of food?’** | | | | | |
| Valued (pre) | 14.79 | 3.91 | 15.81 | 3.24 | *t*(62) = -1.13, *p =* .26 |
| Valued (post) | 13.61 | 4.33 | 13.48 | 4.08 | *t*(62) = 0.12, *p =* .91 |
| Devalued (pre) | 13.97 | 4.13 | 14.74 | 3.57 | *t*(62) = -0.80, *p =* .43 |
| Devalued (post) | 13.88 | 5.08 | 14.94 | 3.89 | *t*(62) = -0.93, *p =* .36 |

*Exploratory gender analysis*

The gender x value interaction and main effect of gender were both *F*<1.

*Experiment 3B*

*Results*

*Hunger ratings.* There were two missing values for hunger before devaluation in the 3-Day group. Hunger ratings decreased from pre- to post-devaluation, *F*(1,47) = 139.3, *p* < .001 (Supplemental Table 3A). There was no main effect of Group, *F*(1,47) = 2.77, *p* = .10, and no interaction between Group and Time, *F* < 1.

*Supplemental Table 3A.* Descriptive statistics for self-report measures by group (1-Day, 3-Day) for Experiment 3A. Pre = pre-devaluation (selective satiety) manipulation; Post = post-devaluation (selective satiety) manipulation. All ratings were completed on Likert scales from 1-7, except for the Hunger ratings which were b=on Likert scales from 1-10.

|  | **1-Day**  **(*N* = 24)** | | **3-Day**  **(*N* = 27)** | |  |
| --- | --- | --- | --- | --- | --- |
|  | ***M*** | ***SD*** | ***M*** | ***SD*** | ***t-tests (two-tailed)*** |
| **‘How hungry are you right now?’** | | | | | |
| Hunger (pre) | 6.21 | 1.32 | 5.68 | 1.70 | *t*(47) =1.21, *p* = .23 |
| Hunger (post) | 3.92 | 1.18 | 3.30 | 1.49 | *t*(49) = 2.12, *p* = .11 |
| **‘How concentrated were you during the task after watching the movies?’** | | | | | |
| Concentration test | 4.96 | 1.23 | 4.37 | 1.23 | *t*(49) = 1.55, *p =* .13 |
| **‘How confident were you that pressing more resulted in more rewards?’** | | | | | |
|  | | | | | |
| Confidence contingency | 5.38 | 1.41 | 3.81 | 1.67 | *t(*49) = -3.59, *p* = .001 |
|  |  |  |  |  |  |
| **‘How confident were you that you would be asked to eat the rewards at the end?’** | | | | | |
| Confidence reward | 6.13 | 1.00 | 5.81 | 1.18 | *t*(49) = 1.01, *p* = .32 |
| **‘How much did you enjoy the task before the movies?’ (very boring 🡪 very enjoyable)** | | | | | |
| Liking training | 3.04 | 1.52 | 2.81 | 1.21 | *t*(49) = .59, *p* = .56 |
| **‘How much did you enjoy the task after the movies?’ (very boring 🡪 very enjoyable)** | | | | | |
| Liking extinction test | 3.46 | 1.32 | 3.07 | 1.27 | *t*(49) = 1.06, *p* = .29 |
|  |  |  |  |  |  |
| **‘How much did you enjoy the task with the arrows?’ (very boring 🡪 very enjoyable)** | | | | | |
| Liking slips-of-action test | 4.33 | .96 | 4.63 | 1.36 | *t*(49) = .89, *p* = .38 |

*Exploratory gender analysis*

As in Experiment 3A, the gender x value interaction and main effect of gender were both *F*<1.

***References***

Carver, C. S., & White, T. L. (1994). Behavioral inhibition, behavioral activation, and affective responses to impending reward and punishment: The BIS/BAS Scales. *Journal of Personality and Social Psychology*, *67*(2), 319–333. http://doi.org/10.1037/0022-3514.67.2.319

Kruschke, J. (2013). Bayesian estimation supersedes the t test. *Journal of Experimental Psychology - General*, *142*, 573–603.

Verhoeven, A., Kindt, M., Zomer, C., & de Wit, S. (2017). An experimental investigation of breaking learnt habits with verbal implementation intentions. *Acta Psychologica*. http://doi.org/10.1016/j.actpsy.2017.05.008
